# Metal–organic framework with optimally selective xenon adsorption and separation

**DOI:** 10.1038/ncomms11831

**Published:** 2016-06-13

**Authors:** Debasis Banerjee, Cory M. Simon, Anna M. Plonka, Radha K. Motkuri, Jian Liu, Xianyin Chen, Berend Smit, John B. Parise, Maciej Haranczyk, Praveen K. Thallapally

**Affiliations:** 1 Physical and Computational Science Directorate, Pacific Northwest National Laboratory, Richland, Washington 99352, USA; 2 Department of Chemical and Biochemical Engineering, University of California, Berkley, Berkeley, California 94720, USA; 3 Department of Geosciences, Stony Brook University, Stony Brook, New York 11794, USA; 4 Energy and Environmental Directorate, Pacific Northwest National Laboratory, Richland, Washington 99352, USA; 5 Department of Chemistry, Stony Brook University, Stony Brook, New York 11794, USA; 6 Institut des Sciences et Ingénierie Chimiques, Valais, Ecole Polytechnique Fédérale de Lausanne (EPFL), Rue de l′Industrie 17, CH-1951 Sion, Switzerland; 7 Photon Sciences, Brookhaven National Laboratory, Upton, New York 11973, USA; 8 Computational Research Division, Lawrence Berkeley National Laboratory, Berkeley, California 94720, USA; 9 IMDEA Materials Institute, C/Eric Kandel 2, 28906 Getafe, Madrid, Spain

## Abstract

Nuclear energy is among the most viable alternatives to our current fossil fuel-based energy economy. The mass deployment of nuclear energy as a low-emissions source requires the reprocessing of used nuclear fuel to recover fissile materials and mitigate radioactive waste. A major concern with reprocessing used nuclear fuel is the release of volatile radionuclides such as xenon and krypton that evolve into reprocessing facility off-gas in parts per million concentrations. The existing technology to remove these radioactive noble gases is a costly cryogenic distillation; alternatively, porous materials such as metal–organic frameworks have demonstrated the ability to selectively adsorb xenon and krypton at ambient conditions. Here we carry out a high-throughput computational screening of large databases of metal–organic frameworks and identify SBMOF-1 as the most selective for xenon. We affirm this prediction and report that SBMOF-1 exhibits by far the highest reported xenon adsorption capacity and a remarkable Xe/Kr selectivity under conditions pertinent to nuclear fuel reprocessing.

## Abstract (web summary)

Increased nuclear energy usage requires the reprocessing of used nuclear fuel to recover radioactive waste, including xenon. Here, the authors perform high-throughput computational screening to identify a metal-organic framework with high xenon selectivity, and demonstrate this with performance analysis.

## Graphical abstract

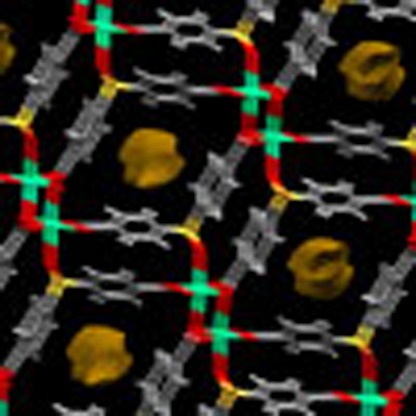

One of the grandest challenges of our generation is to meet our rapidly growing energy demand without further increasing the emission of greenhouse gases^1^,^2^. Nuclear energy is one of the cheapest alternatives to carbon-based fossil fuels that, because of its high energy density and minimal land use requirements, can be scaled up to meet global energy demands. Life cycle analyses indicate that greenhouse gas emissions of a nuclear power plant are significantly lower than fossil fuel technologies and comparable to other renewable electricity generation technologies, such as solar photovoltaics^3^. For the mass implementation of nuclear energy as a low-emissions energy source, we must also safely sequester the associated high-level radioactive waste^2^. In this, most attention is given to recovering the heavy, long-lived nuclear elements in used nuclear fuel (UNF), such as uranium and plutonium. Less discussed are the volatile radionuclides (for example, Xe, Kr) that evolve into the off-gas of UNF aqueous reprocessing facilities^4^. In these off-gases, gaseous radioactive ^85^Kr has a long half-life (*t*_1/2_=10.8 years) and therefore must be captured and removed from the off-gas to prevent its uncontrolled release into the atmosphere^4^. In contrast, the radioactive Xe isotopes (*t*_1/2_≈36.3 days for ^127^Xe) have decayed by the time the fuel is reprocessed. As high purity Xe is used in many applications, including commercial lighting, propulsion, imaging, anesthesia and insulation, the recovered Xe could be sold into the chemical market to offset operating costs. At present, cryogenic distillation is the most mature technology to separate Xe and Kr from air, but it is energy- and capital-intensive and therefore expensive^5^,^6^. Furthermore, the radiolytic formation of ozone poses an explosion hazard during cryogenic distillation^4^. These factors incentivize the development of an alternative technology for a less energy-intensive, more cost-effective and safer process to capture Kr and Xe from UNF reprocessing facility off-gas.

A promising alternative technology for Xe/Kr removal from reprocessing off-gas is an adsorption-based process at room temperature using a selective, solid-state adsorbent. These solid-state adsorbents are found to be almost exclusively Xe-selective, and thus a dual step process whereby, first, the Xe is selectively removed from the off-gas, is a necessary requirement for a practical application^7^. In the subsequent step, the radioactive Kr can be removed from the Xe-free effluent using the same material or a different material. Adsorbents such as silver-loaded zeolites and activated carbon have been proposed^4^, but these fall short compared with high surface area, crystalline metal–organic frameworks (MOFs) and porous organic cage compounds^8^,^9^,^10^,^11^,^12^,^13^,^14^,^15^,^16^,^17^,^18^,^19^,^20^,^21^,^22^. Among the many novel materials tested thus far, HKUST-1 (ref. 14), Co-formate^15^,^20^ and CC3 (ref. 12) are shown to be promising for Xe/Kr separations, showing high capacity and good selectivity for Xe over Kr.

An important advantage of MOFs is their chemical tunability; by combining different linkers and metal centres that self-assemble to form ordered, pre-determined crystal structures, one can synthesize millions of possible materials^23^. MOFs can thus be tailor-made to be optimal for applications related to gas storage and separation, catalysis, chemical sensing and optics^9^,^11^,^23^,^24^,^25^,^26^,^27^,^28^,^29^,^30^,^31^,^32^,^33^,^34^. Our goal here is to identify an optimal MOF for selectively capturing Xe from the off-gas of UNF reprocessing facilities. In practice, however, constraints in resources allow us to synthesize and test only a small subset of chemical space. Molecular models and simulations of adsorption can rapidly and cost-effectively rank MOFs by their Xe/Kr selectivity with reasonable accuracy (Supplementary Methods). High-throughput computational screenings thus play a valuable role of elucidating design rules, determining performance limits, and predicting performance rankings of materials to focus experimental efforts on the most promising MOFs for Xe/Kr separations^35^,^36^,^37^,^38^.

In this work, we use molecular simulations to screen over 125,000 MOF structures^39^,^40^ for selectively adsorbing Xe over Kr at dilute conditions pertinent to UNF reprocessing. Our computational screening predicts that one of the most Xe-selective MOFs is a calcium-based nanoporous MOF, SBMOF-1 [also known as CaSDB, SDB=4,4 -sulfonyldibenzoate], that has not yet been tested for Xe/Kr separations^41^. We affirm this prediction by synthesizing SBMOF-1 and measuring its pure-component Xe and Kr adsorption isotherms. SBMOF-1 exhibits the highest Xe Henry coefficient and thermodynamic Xe/Kr selectivity at dilute conditions among MOFs tested to date. In addition to its high thermal and chemical stability, column breakthrough experiments reveal that SBMOF-1 is a practical, near-term material for capturing Xe from reprocessing facilities.

## Results

### High-throughput computational screening

**Figure 1:**
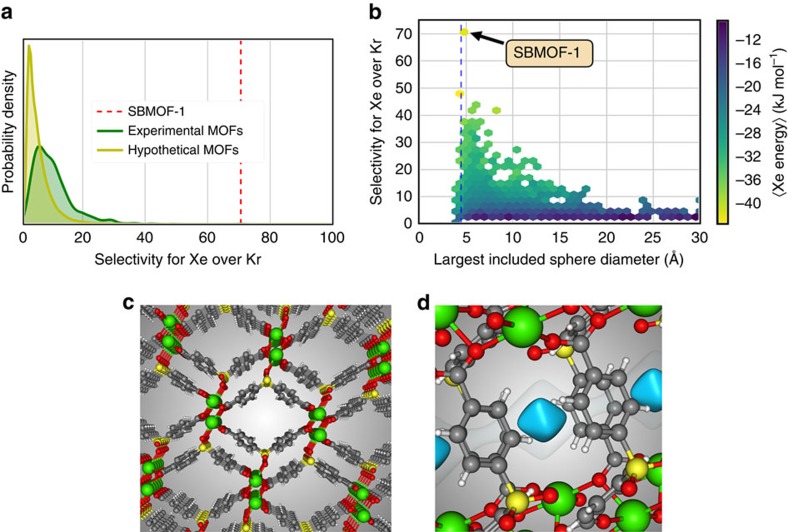
Computational screening of MOFs for Xe/Kr separations at dilute conditions relevant to UNF reprocessing off-gas. We computed the Henry coefficients of Xe and Kr in ∼125,000 MOF structures; the selectivity at dilute conditions is the ratio of Henry coefficients. (**a**) Distribution of simulated selectivities for experimentally synthesized (green) and hypothetical (yellow) MOF structures; vertical, dashed line is SBMOF-1 (KAXQIL in the Cambridge Structural Database (CSD)). (**b**) Histogram showing relationship between selectivity and pore size, with the largest included sphere diameter as a metric; colour shows average energy of Xe adsorption in that bin. SBMOF-1 (KAXQIL in CSD), with simulated selectivity 70.6 and largest included sphere diameter of 5.1 Å, is indicated. Vertical, dashed line is the distance that yields the minimum energy in a Xe–Xe Lennard–Jones potential. (**c**) SBMOF-1 is composed of corner sharing, octahedrally coordinated calcium chains along the crystallographic *b* direction, which are connected by organic linkers, forming a one-dimensional nanoporous channel. (**d**) Side view. Shown are the calculated potential energy contours of a Xe atom adsorbed in the pore (blue surface, −32 kJ mol^−1^; white surface, 15 kJ mol^−1^).

For capturing Xe from nuclear reprocessing, the Xe/Kr selectivity is the most important thermodynamic property determining the performance of a MOF. We used molecular simulations to predict the Xe/Kr selectivity of 125,000 MOF structures at dilute conditions relevant to UNF reprocessing (Supplementary Methods, Fig. 1a,b). The distribution of simulated selectivities in the MOFs is shown in Fig. 1a. We partitioned this distribution into a database of existing MOFs (∼5,000 structures)^39^ and a database of predicted/hypothetical structures (∼120,000)^40^. These distributions span a large range of selectivities, illustrating the unique tunability of MOF materials. Our simulations predict that the most selective material in the database of existing MOFs is SBMOF-1 (Fig. 1c), a three-dimensional, permanently porous MOF (Cambridge Structural Database (CSD) code: KAXQIL)^41^. Furthermore, the Xe/Kr selectivity of SBMOF-1 is ranked in the top 0.01 percentile in the database of 120,000 hypothetical MOFs (Fig. 1b). The red line in Fig. 1b illustrates the outlying Xe/Kr selectivity of SBMOF-1 predicted by our screening. While SBMOF-1 has been synthesized and considered for CO_2_/N_2_ separation^41^, it has not been tested for Xe/Kr separations.

### Synthesis and equilibrium adsorption measurements

**Figure 2:**
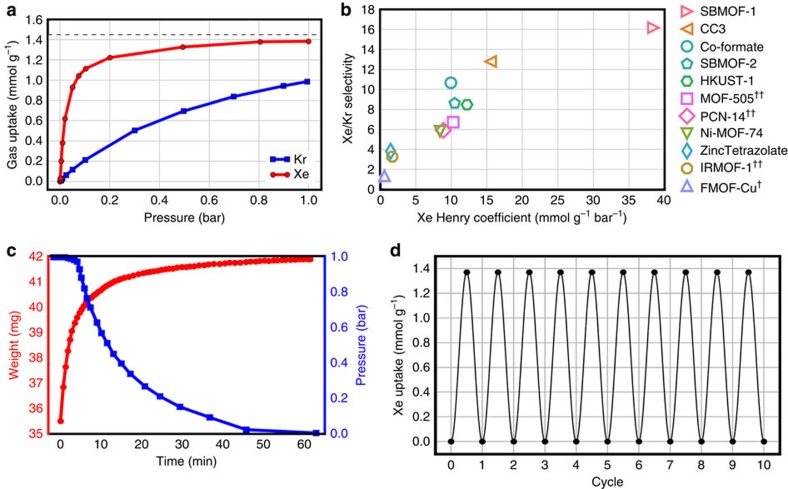
Experimental characterization of Xe and Kr adsorption in SBMOF-1. (**a**) Experimental Xe and Kr adsorption isotherms. Horizontal line indicates one atom per pore segment. (**b**) Survey of thermodynamic Xe/Kr separation performance in top-performing materials. Henry coefficients are extracted from pure-component Xe and Kr adsorption isotherms reported in the literature (see Methods). Data at 298 K, exceptions denoted by a dagger (†) for 297 K and a double dagger (††) for 292 K. (**c**) Xe adsorption kinetics experiments. The blue curve shows the pressure drop in a chamber feeding Xe to an initially evacuated chamber with the SBMOF-1 sample; the red curve shows the corresponding weight increase due to Xe adsorption. (**d**) Xe adsorption/desorption cycling data; a sinusoidal curve is superimposed on the data. (**a**,**c**,**d**) Data for SBMOF-1 at 298 K.

Encouraged by the data from our high-throughput screening, we synthesized SBMOF-1 and measured its pure-component Xe and Kr adsorption isotherms at room temperature (see synthesis section of Supplementary Methods, Supplementary Figs 17–21)^41^. Our first measurement of low pressure Xe uptake in SBMOF-1 at 298 K, when activated by the reported activation procedure^41^, was much lower than predicted by molecular simulation (Supplementary Fig. 22). However, we found that activating SBMOF-1 at a lower temperature yielded low pressure Xe uptake closer to the simulation (Fig. 2a, Supplementary Fig. 23, see effect of activation temperature section of the Supplementary Methods). The Xe adsorption isotherm in SBMOF-1 saturates at a low pressure, indicative of a high affinity for Xe compared with other gases including Kr (Fig. 2a, Supplementary Figs 24 and 25). The Kr adsorption isotherm exhibits a smaller slope and does not saturate even at 1 bar, indicative of a much weaker affinity for Kr. This hints that SBMOF-1 is highly discriminatory for Xe over Kr. Indeed, identifying the Xe and Kr Henry coefficients from the pure-component adsorption isotherms, we predict SBMOF-1 to exhibit a thermodynamic Xe/Kr selectivity of 16 at dilute conditions at 298 K.

It is interesting to compare the equilibrium Xe and Kr uptake of SBMOF-1 with the reported top-performing MOFs. We collected from the literature experimentally measured Xe and Kr adsorption isotherms in Co-formate^15^,^20^, SBMOF-2 (ref. 13), HKUST-1 (ref. 14), MOF-505 (ref. 10), PCN-14 (ref. 19), Ni-MOF-74 (ref. 8), Zinc tetrazolate^21^, IRMOF-1 (ref. 22), and FMOF-Cu^9^ and identified the Xe and Kr Henry coefficients from the data in the low pressure regime (see Computational Calculation section of Supplementary Methods, Fig. 2b, Supplementary Figs 1–16). The saturation loading of Xe in SBMOF-1 is lower than observed in the majority of these materials due to the comparatively low (∼145 m^2^ g^−1^) surface area of SBMOF-1 (Supplementary Figs 26 and 27)^41^. However, the Henry coefficient of Xe in SBMOF-1 is a factor of two higher than in CC3, the material in our survey with the second highest Xe Henry coefficient; we thus expect SBMOF-1 to have an outstanding Xe uptake under UNF reprocessing off-gas conditions. Figure 2b shows that SBMOF-1 exhibits by far the largest Xe Henry coefficient and the highest Xe/Kr selectivity at dilute conditions among all reported Xe and Kr adsorption isotherms in our literature survey.

### Adsorption kinetics and column breakthrough experiments

**Figure 3:**
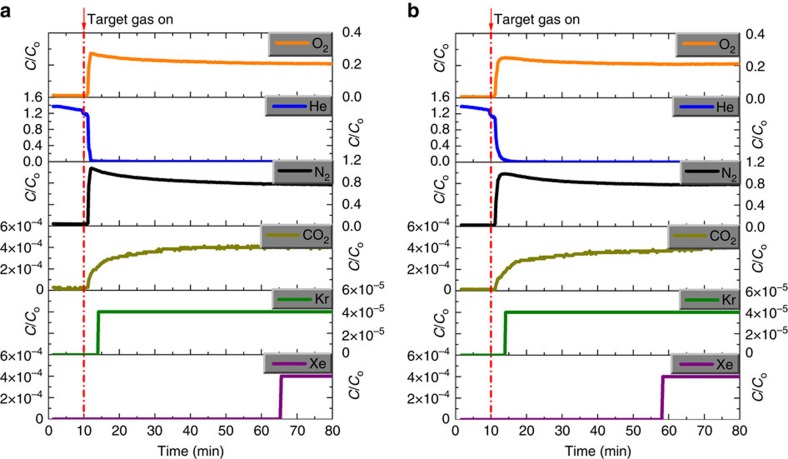
Single column breakthrough experiments using SBMOF-1 at room temperature and 1 atm. Column is initially purged with He. (**a**) Inlet is a dry gas mixture with 400 p.p.m. Xe and 40 p.p.m. Kr balanced with air. (**b**) Inlet is the same gas mixture as in (**a**) with 42% relative humidity. Note that the Xe breakthrough time is only marginally decreased in the presence of water.

From a practical point of view, it is important that the kinetics of Xe adsorption/desorption are sufficiently fast and the material can undergo multiple ad-/de-sorption cycles without losing capacity. We measured the kinetics of Xe adsorption into an SBMOF-1 sample by connecting a chamber of Xe at 1 bar and 298 K to an evacuated chamber with the SBMOF-1 sample, then opening a valve to allow flow. Figure 2c shows that the rate of Xe uptake is sufficiently fast, reaching ∼80% of saturation uptake within 10 min. Next, we performed 10 ad-/de-sorption cycles to test if SBMOF-1 retains its high Xe adsorption capacity after many cycles. Figure 2d shows that SBMOF-1 retains its performance after multiple cycles. In addition, SBMOF-1 shows high thermal stability up to 500 K (Supplementary Fig. 20). To demonstrate the practical applicability of SBMOF-1 for capturing Xe from UNF reprocessing off-gas, we conducted single-column breakthrough experiments with a representative gas mixture (400 p.p.m. Xe, 40 p.p.m. Kr, 78.1% N_2_, 20.9% O_2_, 0.03% CO_2_ and 0.9% Ar) (see breakthrough measurement section of the Supplementary Information, Supplementary Figs 28 and 29)^17^. We fed this gas mixture through a column packed with SBMOF-1 and initially purged with He. Figure 3 shows that all gases except Xe broke through the column within minutes, whereas Xe was retained in the column for more than an hour). This demonstrates that SBMOF-1 can selectively remove Xe from air at UNF reprocessing conditions. Under these conditions, SBMOF-1 adsorbed 13.2 mmol Xe per kg, higher than the reported breakthrough Xe capacities of benchmark materials, Ni-MOF-74 (4.8 mmol Xe per kg) and CC3 (11 mmol Xe per kg) (Supplementary Fig. 29)^12^,^17^. The experimental breakthrough capacity is close to that predicted from the Henry coefficient of the pure-component Xe isotherm (15.4 mmol kg^−1^), suggesting minimal diffusion limitations in the SBMOF-1 pellets. Next, we conducted column breakthrough experiments on SBMOF-1 in the presence of 42% relative humidity (Fig. 3b). Remarkably, SBMOF-1 retains a high Xe uptake (∼11.5 mmol kg^−1^) even in the presence of water vapor. These results suggest the outstanding stability of SBMOF-1 makes it a practical material for the removal of Xe from UNF reprocessing off-gas. Such stability is a desirable property, as very few metal–organic hybrid materials exhibit such properties^42^,^43^,^44^. We postulate the absence of open metal sites to be responsible for the stability of SBMOF-1 in the presence of water vapour^45^.

### Revealing the Xe adsorption sites in SBMOF-1

**Figure 4:**
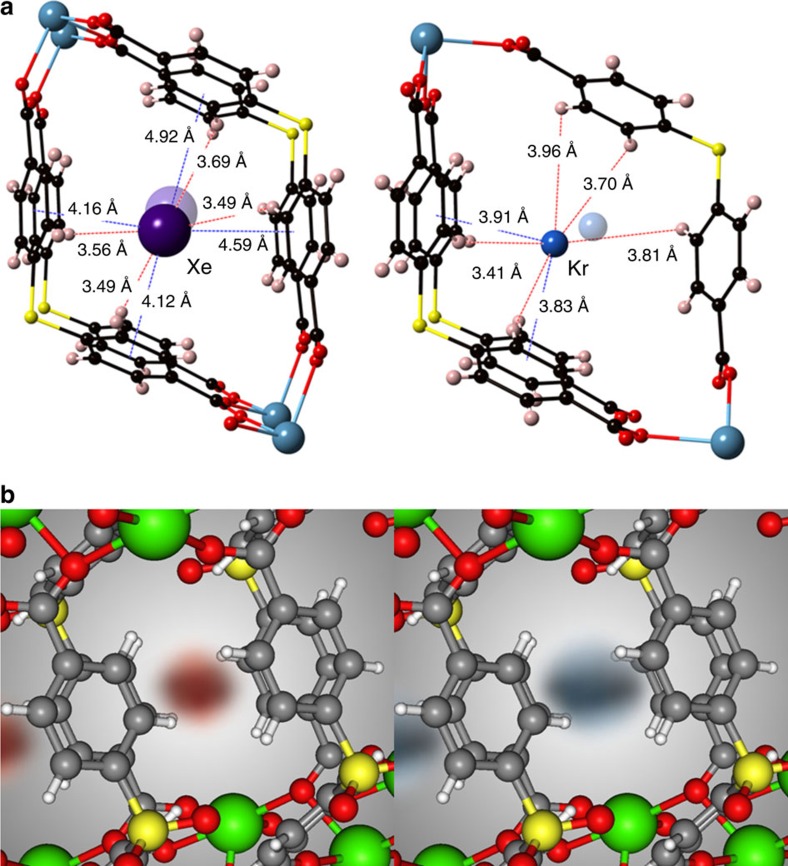
Xe and Kr adsorption sites in SBMOF-1. (**a**) Xe and Kr positions determined by single-crystal X-ray diffraction. (**b**) Spatial probability densities from recording adsorbate positions during pure-component grand-canonical Monte Carlo simulations at 1 bar and 298 K (Xe, red; Kr, blue).

To identify the location of adsorbed Xe and Kr, we performed single-crystal X-ray diffraction experiments on activated SBMOF-1 (Supplementary Data 1 and 2). Single-crystal analysis of Xe-loaded SBMOF-1 reveals that Xe adsorbs at a single site, near the midpoint of the channel, interacting with the channel wall composed of aromatic rings by mainly van der Waals interactions. Due to symmetry considerations (space group *P*2_1_/c), each Xe atom is positioned at two possible sites (Fig. 4, Supplementary Table 1). The distance between each Xe atom along the *b* axis is 5.56 Å, closely matching the *b* axis length of the unit cell. There are 1.72 atoms of Xe per unit cell based on crystallographic analysis (∼1.25 mmol g^−1^), close to the loading obtained from gas-adsorption data (1.38 mmol g^−1^). The saturation loading of Xe in Fig. 2a approaches two atoms per unit cell (see horizontal line), indicating commensurate Xe adsorption, which occurs when the adsorbed amount, location and orientation of an adsorbate are commensurate with the crystallographic symmetry of the adsorbent^46^. Such commensurate adsorption in SBMOF-1 was previously observed for small hydrocarbon molecules (C_2_–C_3_)^47^,^48^. The observed position of Xe in the pore is consistent with calculated potential energy contours and molecular simulations of Xe adsorption (Figs 1d and 4b, Supplementary Fig. 30, Supplementary Table 2).

We can rationalize the high Xe adsorption capacity and selectivity exhibited by SBMOF-1 by its optimal Xe adsorption site. First, the pore size of SBMOF-1 is tailored for Xe^35^,^38^. As a metric for pore size, we calculate the diameter of the largest included hard-sphere that can fit inside the pore of SBMOF-1 as 4.2 Å, slightly larger than a Xe atom, ∼4.1 Å. Simulations of Xe/Kr adsorption in the database of experimental MOFs show that all of the most selective MOFs have pore sizes slightly larger than a Xe atom (Fig. 1b). Such a pore diameter is a prerequisite for a highly Xe-selective material, as the pore size controls the proximity and degree of overlap from multiple framework atoms contributing van der Waals interactions from multiple directions to achieve a highly favourable host–Xe interaction. A pore of optimal size for Xe is suboptimal for Kr because of the size difference, so this forms a pore that is highly discriminatory for Xe over Kr^38^. As shown in Fig. 1c, the pore size of SBMOF-1 falls in the optimal pore size window for Xe/Kr separations^37^, distinguishing it from other MOFs. Porous organic cage CC3, another outstanding Xe-selective material, also exhibits a pore size tailored for Xe (pore window 4.4 Å), but SBMOF-1 constructs a denser wall of chemical moieties than CC3 to achieve a higher Xe binding energy, enhancing its preference for Xe (Supplementary Table 3). This is the second reason why SBMOF-1 is outstanding in Xe adsorption; the colour in Fig. 1b shows that the dense wall of SBMOF-1 surrounds a Xe atom to achieve a high energy of Xe adsorption and thus a high Xe selectivity, following the trend in other MOFs.

## Discussion

We demonstrated that a nanoporous MOF, SBMOF-1, identified as an outstanding Xe/Kr selective material from molecular simulations, shows exceptional Xe uptake at low pressure, selectivity for Xe, thermal and water stability, and adsorption kinetics. These attributes make SBMOF-1 potentially useful as a practical, near-term material for removal of Xe and Kr from nuclear reprocessing facilities with a far less energy requirement than cryogenic distillation. The selective adsorption of Xe from relevant gas mixtures even with ∼42% relative humidity demonstrate practicality and offer improvements over current technologies. Our recent economic analysis showed the cost benefits of using Ni-MOF-74 for an adsorption-based separation process at room temperature in comparison with cryogenic distillation^7^,^11^. The discovery of the high Xe uptake and selectivity of SBMOF-1 at UNF reprocessing conditions—also in the presence of humidity—will enable an even more cost-effective process. The exceptional selectivity of SBMOF-1 is attributed to its pore size tailored to Xe and its dense wall of atoms that constructs a binding site with a high affinity for Xe, as evident by single-crystal X-ray diffraction and molecular models. As molecular simulations predicted SBMOF-1 to be among the most selective of ∼5,000 experimentally reported MOFs and ∼120,000 hypothetical MOF structures *a priori*, this work is a rare case of a computationally inspired materials discovery.

## Methods

### Synthesis and scale up

SBMOF-1 was originally synthesized using a previously published literature procedure^41^. In a typical synthesis, a mixture of 0.6 mmol of CaCl_2_ (0.074 g) and 0.6 mmol of 4, 4′-SDB (0.198 g) were added in 10 ml of ethanol and stirred for ∼2 h to achieve homogeneity (molar ratio of metal chloride:ligand:solvent=1:1:380). The resultant solution was heated at 180 °C for 3 days. Colourless, needle-shaped crystals were recovered as product and washed with ethanol (yield: 45% based on CaCl_2_, 0.1 g). For scale up, 1.44 g of CaCl_2_ (13 mmol) and 3.98 g of 4,4′-SDB (13 mmol) were added to 120 ml of ethanol and stirred for ∼2 h to achieve homogeneity (molar ratio: 1:1:156). The well-mixed solution was then transferred to three 100 ml Teflon-lined stainless steel Parr autoclaves and heated for 3 days at 180 °C. The product was obtained as white powder and washed by ethanol (3 times, 50 ml), followed by drying under vacuum (yield: 2.2 g, 50% based on CaCl_2_). The as-synthesized material was then exchanged with methanol (3 × , 50 ml) for a total period of 3 days. The product purity was confirmed by powder XRD.

### Gas-adsorption and breakthrough experiments

The methanol-exchanged SBMOF-1 was activated at 100 °C for 12 h under dynamic vacuum. Single-component gas-adsorption isotherms were collected in a Quantachrome Autosorb-1 and dynamic sorption analyzer (ARBC, Hiden Analytical Ltd., Warrington, UK). The later instrument was also used to collect breakthrough measurement data. Breakthrough measurements were conducted on 20–35 mesh (500–850 μm) pellets of SBMOF-1 (1.48 g) using a gas mixture composition simulating UNF conditions (400 p.p.m. Xe, 40 p.p.m. Kr, 78.1% N_2_, 20.9% O_2_, 0.03% CO_2_ and 0.9% Ar).

### Computational methodologies

At dilute conditions relevant to UNF reprocessing off-gas, we modelled Xe and Kr adsorption in the MOFs with Henry's law. Let *P* be the pressure (units: bar) and *σ*(*P*) be the gas uptake (units: mmol g^−1^) as a function of pressure (the adsorption isotherm). Henry's law, only valid at low surface coverage, is then:


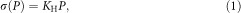


where *K*_H_ is the Henry coefficient (units: mmol g^−1^ bar^−1^) of the gas in the adsorbent. The Xe/Kr selectivity is then the ratio of the Henry coefficients. We calculated the Henry coefficient in each MOF using Widom particle insertions, a Monte Carlo integration^49^. We model the energetic interactions between Xe and Kr with the atoms of the MOFs using Lennard Jones potentials. We took parameters for Xe and Kr from Boato *et al.* and for the MOF atoms from the Universal Force Field, applying Lorentz–Berthelot mixing rules to obtain cross-interactions^50^,^51^. We hold the MOF structures rigid throughout the simulation and apply periodic boundary conditions to mimic an infinite crystal. For potential energy contours and spatial probability density plots for SBMOF-1, we utilized a hybrid Dreiding-TraPPE force field, as this force field produces a better match to the Xe and Kr isotherms in SBMOF-1 than the UFF^52^,^53^. The largest included hard sphere diameter is calculated using Zeo++ (refs 54, 55). See Supplementary Methods for more details.

### Literature survey for pure-component Xe and Kr adsorption isotherms

To generate Fig. 2b, we collected from the literature experimentally measured single-component Xe and Kr adsorption isotherms in MOFs and porous organic cage materials. Focusing on the low-pressure regime of the adsorption isotherm that exhibits linear behaviour—the Henry regime where Henry's law in equation (1) is valid—we fit a line with zero intercept to this data to identify *K*_H_ of Xe and Kr in the material. See Supplementary Section for the data and visualizations of the resulting fits to equation (1). Our data and code to reproduce Fig. 2b are openly available on GitHub at https://github.com/CorySimon/XeKrMOFAdsorptionSurvey.

### Single-crystal X-ray diffraction

The single-crystal data on the Xe- and Kr-loaded activated SBMOF-1 were collected using a four circle kappa Oxford Gemini diffractometre equipped with an Atlas detector (*λ*=0.71073) at 100 K. The raw intensity data were collected, integrated and corrected for absorption effects using CrysAlis PRO software. Data sets were corrected for absorption using a multi-scan method, and structures were solved by direct methods using SHELXS-97 and refined by full-matrix least squares on *F*^2^ with SHELXL-97 (ref. 56).

### Data availability

The X-ray crystallographic coordinates for structures reported in this study have been deposited at the Cambridge Crystallographic Data Centre (CCDC), under deposition numbers 1475229-1475230. These data can be obtained free of charge from the Cambridge Crystallographic Data Centre via www.ccdc.cam.ac.uk/data_request/cif. Our data and code to reproduce Fig. 2b are openly available on GitHub at https://github.com/CorySimon/XeKrMOFAdsorptionSurvey. All other data, if not included in the Article or the Supplementary Information, are available from the authors on request.

## Additional information

**How to cite this article:** Banerjee, D. *et al.* Metal–organic framework with optimally selective xenon adsorption and separation. *Nat. Commun.* 7:11831 doi: 10.1038/ncomms11831 (2016).

## Supplementary Material

Supplementary Information Supplementary Figures 1-30, Supplementary Tables 1-3 and Supplementary Methods

Supplementary Data 1 Crystallographic Information File for Xe-containing MOF

Supplementary Data 2 Crystallographic Information File for Kr-containing MOF

## References

[b1] Hoffert M. I. *et al.* Advanced technology paths to global climate stability: energy for a greenhouse planet. Science 298, 981–987 (2002). 12411695 10.1126/science.1072357

[b2] Chu S. & Majumdar A. Opportunities and challenges for a sustainable energy future. Nature 488, 294–303 (2012). 22895334 10.1038/nature11475

[b3] Lenzen M. Life cycle energy and greenhouse gas emissions of nuclear energy: a review. Energ. Convers. Manage. 49, 2178–2199 (2008).

[b4] Soelberg N. R. *et al.* Radioactive iodine and krypton control for nuclear fuel reprocessing facilities. Sci. Technol. Nucl. Ins. 2013, 1–12 (2013).

[b5] Ying R. T. Gas Separation by Adsorption Processes Butterworth-Heinemann (2013).

[b6] Kerry F. G. Industrial Gas Handbook: Gas Separation and Purification CRC Press (2007).

[b7] Liu J., Fernandez C. A., Martin P. F., Thallapally P. K. & Strachan D. M. A two-column method for the separation of Kr and Xe from process off-gases. Ind. Eng. Chem. Res. 53, 12893–12899 (2014).

[b8] Thallapally P. K., Grate J. W. & Motkuri R. K. Facile xenon capture and release at room temperature using a metal-organic framework: a comparison with activated charcoal. Chem. Commun. (Camb) 48, 347–349 (2012). 21956410 10.1039/c1cc14685h

[b9] Fernandez C. A., Liu J., Thallapally P. K. & Strachan D. M. Switching Kr/Xe selectivity with temperature in a metal-organic framework. J. Am. Chem. Soc. 134, 9046–9049 (2012). 22591325 10.1021/ja302071t

[b10] Bae Y. S. *et al.* High xenon/krypton selectivity in a metal-organic framework with small pores and strong adsorption sites. Micropor. Mesopor. Mater. 169, 176–179 (2013).

[b11] Banerjee D. *et al.* Potential of metal-organic frameworks for separation of xenon and krypton. Acc. Chem. Res. 48, 211–219 (2014). 25479165 10.1021/ar5003126

[b12] Chen L. *et al.* Separation of rare gases and chiral molecules by selective binding in porous organic cages. Nat. Mater. 13, 954–960 (2014). 25038731 10.1038/nmat4035

[b13] Chen X. *et al.* Direct observation of Xe and Kr adsorption in a Xe-selective microporous metal–organic framework. J. Am. Chem. Soc. 137, 7007–7010 (2015). 26000710 10.1021/jacs.5b02556

[b14] Hulvey Z. *et al.* Noble gas adsorption in copper trimesate, HKUST-1: an experimental and computational study. J. Phys. Chem. C 117, 20116–20126 (2013).

[b15] Lawler K. V., Hulvey Z. & Forster P. M. Nanoporous metal formates for krypton/xenon separation. Chem. Commun. (Camb) 49, 10959–10961 (2013). 24131928 10.1039/c3cc44374d

[b16] Liu J., Strachan D. M. & Thallapally P. K. Enhanced noble gas adsorption in Ag@MOF-74Ni. Chem. Commun. (Camb) 50, 466–468 (2014). 24256738 10.1039/c3cc47777k

[b17] Liu J., Thallapally P. K. & Strachan D. Metal-organic frameworks for removal of Xe and Kr from nuclear fuel reprocessing plants. Langmuir 28, 11584–11589 (2012). 22799439 10.1021/la301870n

[b18] Meek S. T., Teich-McGoldrick S. L., Perry J. J., Greathouse J. A. & Allendorf M. D. Effects of polarizability on the adsorption of noble gases at low pressures in monohalogenated isoreticular metal-organic frameworks. J. Phys. Chem. C 116, 19765–19772 (2012).

[b19] Perry J. J. *et al.* Noble gas adsorption in metal-organic frameworks containing open metal sites. J. Phys. Chem. C 118, 11685–11698 (2014).

[b20] Wang H. *et al.* The first example of commensurate adsorption of atomic gas in a MOF and effective separation of xenon from other noble gases. Chem. Sci. 5, 620–624 (2014).

[b21] Xiong S. S. *et al.* A flexible zinc tetrazolate framework exhibiting breathing behaviour on xenon adsorption and selective adsorption of xenon over other noble gases. J. Mater. Chem. A 3, 10747–10752 (2015).

[b22] Mueller U. *et al.* Metal-organic frameworks - prospective industrial applications. J. Mater. Chem. 16, 626–636 (2006).

[b23] Yaghi O. M. *et al.* Reticular synthesis and the design of new materials. Nature 423, 705–714 (2003). 12802325 10.1038/nature01650

[b24] Zhou H.-C., Long J. R. & Yaghi O. M. Introduction to metal–organic frameworks. Chem. Rev. 112, 673–674 (2012). 22280456 10.1021/cr300014x

[b25] Eddaoudi M., Sava D. F., Eubank J. F., Adil K. & Guillerm V. Zeolite-like metal-organic frameworks (ZMOFs): design, synthesis, and properties. Chem. Soc. Rev. 44, 228–249 (2015). 25341691 10.1039/c4cs00230j

[b26] Ferey G. Hybrid porous solids: past, present, future. Chem. Soc. Rev. 37, 191–214 (2008). 18197340 10.1039/b618320b

[b27] James S. L. Metal-organic frameworks. Chem. Soc. Rev. 32, 276–288 (2003). 14518181 10.1039/b200393g

[b28] Kitagawa S., Kitaura R. & Noro S.-i. Functional porous coordination polymers. Angew. Chem. Int. Ed. 43, 2334–2375 (2004). 10.1002/anie.200300610 15114565

[b29] Li J.-R., Kuppler R. J. & Zhou H.-C. Selective gas adsorption and separation in metal-organic frameworks. Chem. Soc. Rev. 38, 1477–1504 (2009). 19384449 10.1039/b802426j

[b30] Sumida K. *et al.* Carbon dioxide capture in metal–organic frameworks. Chem. Rev. 112, 724–781 (2012). 22204561 10.1021/cr2003272

[b31] Lee J. *et al.* Metal-organic framework materials as catalysts. Chem. Soc. Rev. 38, 1450–1459 (2009). 19384447 10.1039/b807080f

[b32] Kreno L. E. *et al.* Metal–organic framework materials as chemical sensors. Chem. Rev. 112, 1105–1125 (2012). 22070233 10.1021/cr200324t

[b33] Allendorf M. D., Bauer C. A., Bhakta R. K. & Houk R. J. T. Luminescent metal-organic frameworks. Chem. Soc. Rev. 38, 1330–1352 (2009). 19384441 10.1039/b802352m

[b34] Motkuri R. K. *et al.* Fluorocarbon adsorption in hierarchical porous frameworks. Nat. Commun. 5, 4368 (2014). 25006832 10.1038/ncomms5368

[b35] Ryan P., Farha O. K., Broadbelt L. J. & Snurr R. Q. Computational screening of metal-organic frameworks for xenon/krypton separation. Aiche J. 57, 1759–1766 (2011).

[b36] Van Heest T., Teich-McGoldrick S. L., Greathouse J. A., Allendorf M. D. & Sholl D. S. Identification of metal-organic framework materials for adsorption separation of rare gases: applicability of ideal adsorbed solution theory (IAST) and effects of inaccessible framework regions. J. Phys. Chem. C 116, 13183–13195 (2012).

[b37] Sikora B. J., Wilmer C. E., Greenfield M. L. & Snurr R. Q. Thermodynamic analysis of Xe/Kr selectivity in over 137 000 hypothetical metal–organic frameworks. Chem. Sci. 3, 2217 (2012).

[b38] Simon C. M., Mercado R., Schnell S. K., Smit B. & Haranczyk M. What are the best materials to separate a xenon/krypton mixture? Chem. Mater. 27, 4459–4475 (2015).

[b39] Chung Y. G. *et al.* Computation-ready, experimental metal-organic frameworks: a tool to enable high-throughput screening of nanoporous crystals. Chem. Mater. 26, 6185–6192 (2014).

[b40] Wilmer C. E. *et al.* Large-scale screening of hypothetical metal-organic frameworks. Nat. Chem. 4, 83–89 (2012). 22270624 10.1038/nchem.1192

[b41] Banerjee D., Zhang Z. J., Plonka A. M., Li J. & Parise J. B. A calcium coordination framework having permanent porosity and high CO2/N-2 selectivity. Cryst Growth Des 12, 2162–2165 (2012).

[b42] Howarth A. J. *et al.* Chemical, thermal and mechanical stabilities of metal–organic frameworks. Nat. Rev. Mater. 1, 15018 (2016).

[b43] Deria P. *et al.* Ultraporous, water stable, and breathing zirconium-based metal–organic frameworks with ftw topology. J. Am. Chem. Soc. 137, 13183–13190 (2015). 26387968 10.1021/jacs.5b08860

[b44] Kalidindi S. B. *et al.* Chemical and structural stability of zirconium-based metal–organic frameworks with large three-dimensional pores by linker engineering. Angew. Chem. Int. Ed. Engl. 127, 223–228 (2015). 10.1002/anie.201406501 PMC4309464 25521699

[b45] Kizzie A. C., Wong-Foy A. G. & Matzger A. J. Effect of humidity on the performance of microporous coordination polymers as adsorbents for CO2 Capture. Langmuir 27, 6368–6373 (2011). 21488612 10.1021/la200547k

[b46] Wu H., Gong Q., Olson D. H. & Li J. Commensurate adsorption of hydrocarbons and alcohols in microporous metal organic frameworks. Chem. Rev. 112, 836–868 (2012). 22257090 10.1021/cr200216x

[b47] Banerjee D. *et al.* Direct structural evidence of commensurate-to-incommensurate transition of hydrocarbon adsorption in a microporous metal organic framework. Chem. Sci. 7, 759–765 (2016). 10.1039/c5sc03685b PMC5952992 29896359

[b48] Plonka A. M. *et al.* Light hydrocarbon adsorption mechanisms in two calcium-based microporous metal organic frameworks. Chem. Mater. 28, 1636–1646 (2016).

[b49] Frenkel D. & Smit B. Understanding Molecular Simulation: From Algorithms to Applications Academic Press (2001).

[b50] Boato G. & Casanova G. A self-consistent set of molecular parameters for neon, argon, krypton and xenon. Physica 27, 571 -& (1961).

[b51] Rappe A. K., Casewit C. J., Colwell K. S., Goddard W. A. & Skiff W. M. Uff a full periodic-table force-field for molecular mechanics and molecular-dynamics simulations. J. Am. Chem. Soc. 114, 10024–10035 (1992).

[b52] Wick C. D., Martin M. G. & Siepmann J. I. Transferable potentials for phase equilibria. 4. United-atom description of linear and branched alkenes and alkylbenzenes. J. Phys. Chem. B 104, 8008–8016 (2000).

[b53] Mayo S. L., Olafson B. D. & Goddard W. A. Dreiding - a generic force-field for molecular simulations. J. Phys. Chem. 94, 8897–8909 (1990).

[b54] Willems T. F., Rycroft C., Kazi M., Meza J. C. & Haranczyk M. Algorithms and tools for high-throughput geometry-based analysis of crystalline porous materials. Micropor. Mesopor. Mater. 149, 134–141 (2012).

[b55] Pinheiro M., Martin R. L., Rycroft C. H. & Haranczyk M. High accuracy geometric analysis of crystalline porous materials. CrystEngComm 15, 7531–7538 (2013).

[b56] Sheldrick G. M. A short history of SHELX. Acta Cryst. A. 64, 112–122 (2008). 18156677 10.1107/S0108767307043930

