# Supplementary material for: Metal–organic framework with optimally selective xenon adsorption and separation
Source: Nat Commun. 2016 Jun 13;7:ncomms11831. doi: 10.1038/ncomms11831 (PMC4909987; doi:10.1038/ncomms11831)
Supplement: Supplementary Information — Supplementary Figures 1-30, Supplementary Tables 1-3 and Supplementary Methods [file ncomms11831-s1.pdf]

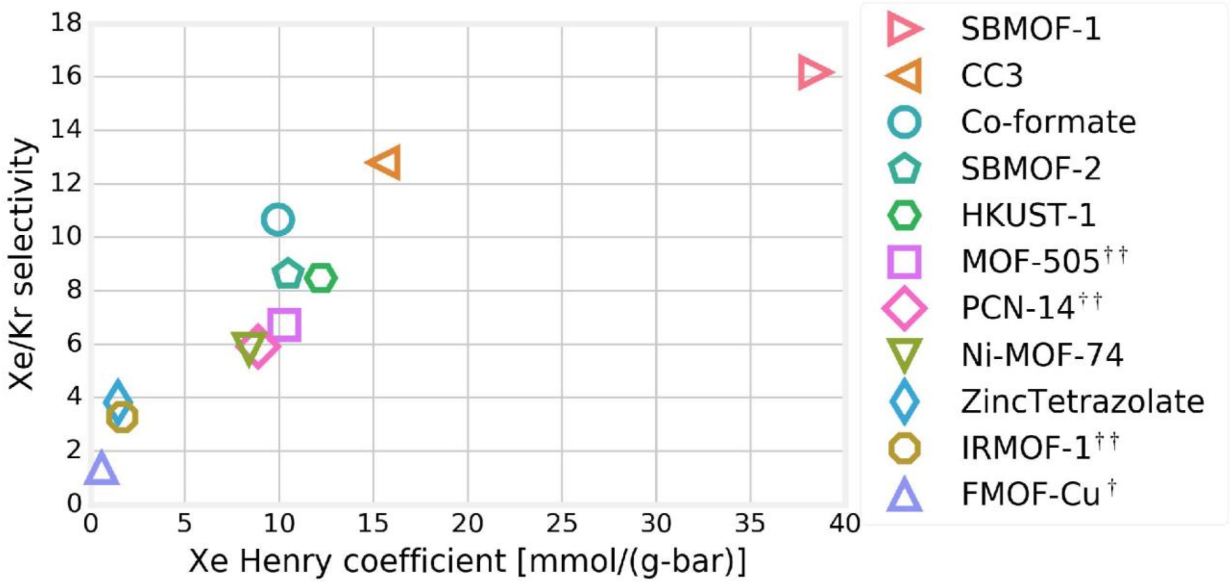

Supplementary Figure 1. Summary of Xe/Kr separation performance of several nanoporous materials on the basis of experimental equilibrium adsorption data. The *y*-axis displays the selectivity for Xe over Kr computed from the ratio of Henry coefficients identified from experimentally measured pure-component Xe and Kr adsorption isotherms. The *x*-axis shows the Xe Henry coefficient. All data is at 298 K, with the exception of MOFs with superscripts in the legend (dagger denotes 297 K, double dagger denotes 292 K).

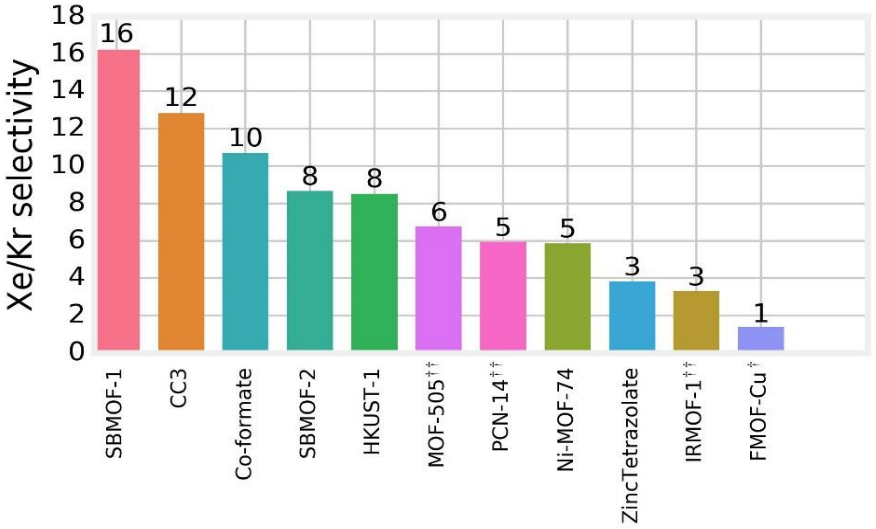

(a)

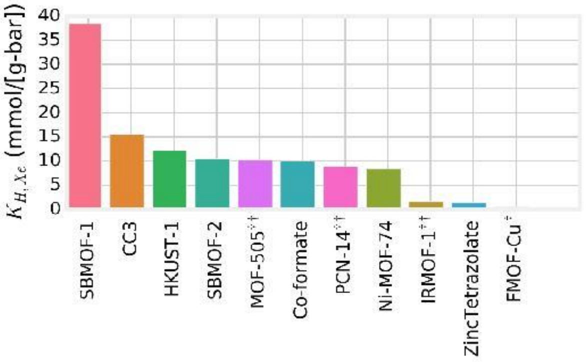

(b)

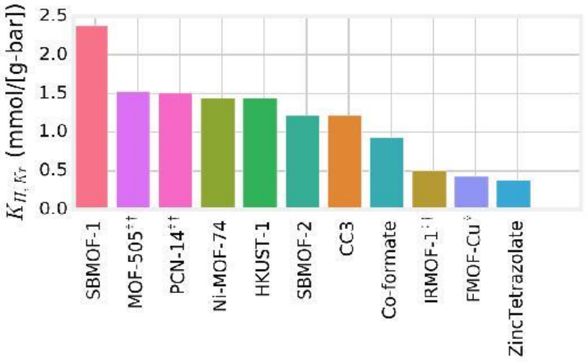

(c)

Supplementary Figure 2. Bar plots showing (a) Xe/Kr selectivity (b) Xe Henry coefficient (c) Kr Henry coefficient. All data is at 298 K, with the exception of MOFs with superscripts in the legend (dagger denotes 297 K, double dagger denotes 292 K).

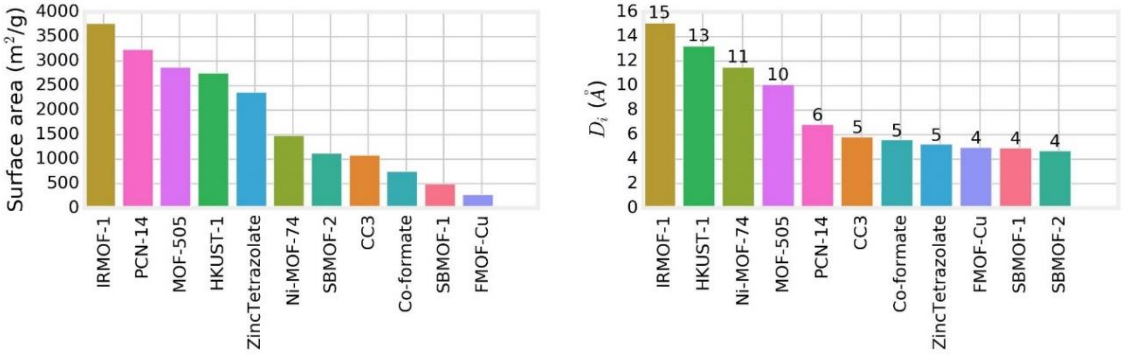

(a) **(b)**

Supplementary Figure 3. Geometric properties calculated from the crystal structures using Zeo++. (a) Surface areas using a 1.25 Å probe to represent helium. (b) Largest included hard-sphere diameters (D_i).

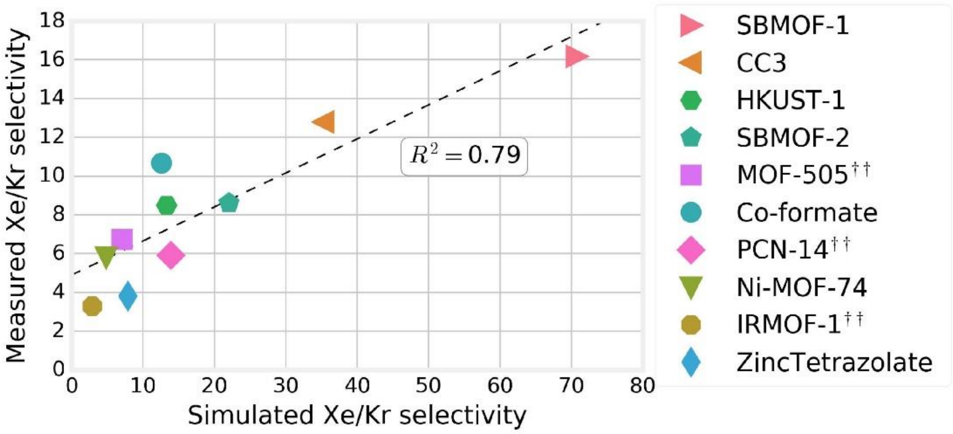

Supplementary Figure 4. Experimental versus simulated Xe/Kr selectivity for materials in this survey. The dashed line displays the line of best fit. (dagger denotes 297 K, double dagger denotes 292 K).

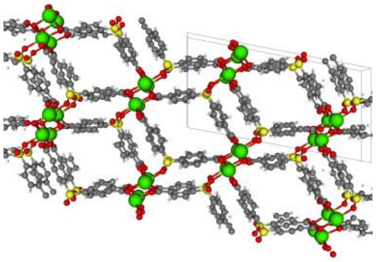

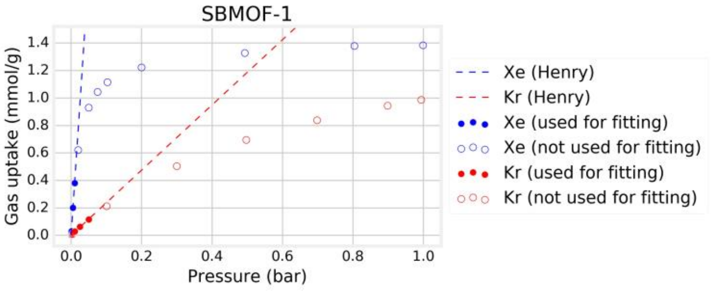

(a) **(b)**[1]

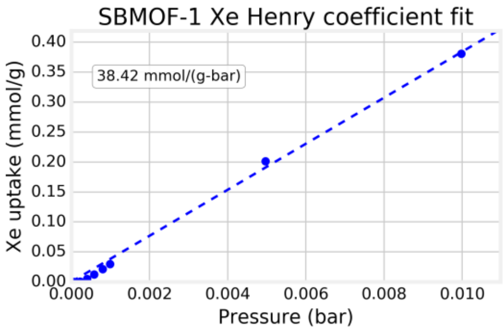

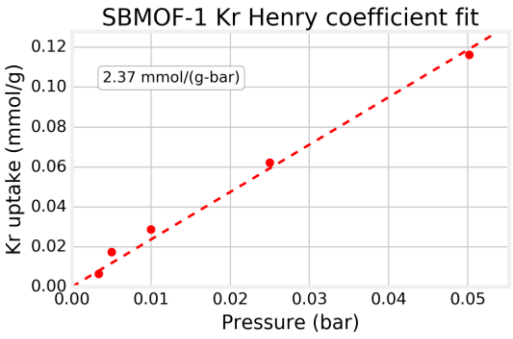

(c) **(d)**

Supplementary Figure 5. SBMOF-1. (a) Crystal structure of SBMOF-1. (b) Circles show pure component Xe (blue) and Kr (red) adsorption isotherm data at 298 K from current work. Closed symbols are data used to identify the Henry coefficient. The dashed line shows Henry's law with the identified Henry coefficient. (c, d) same as (b) but zoomed into the Henry regime. Identified coefficient is shown in the box.

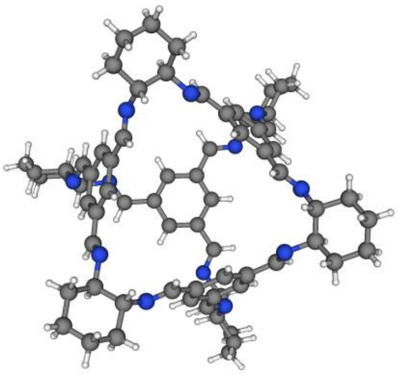

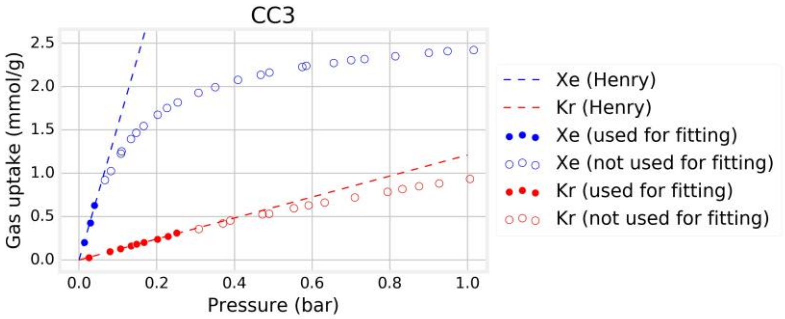

(a) **(b)**

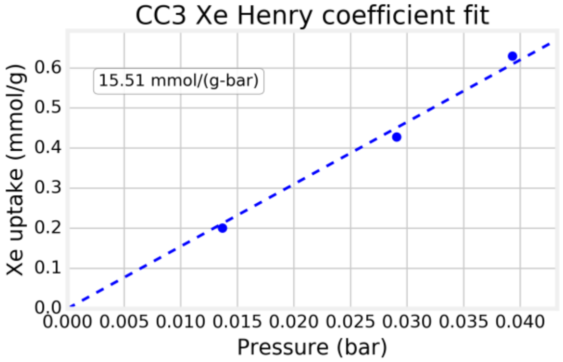

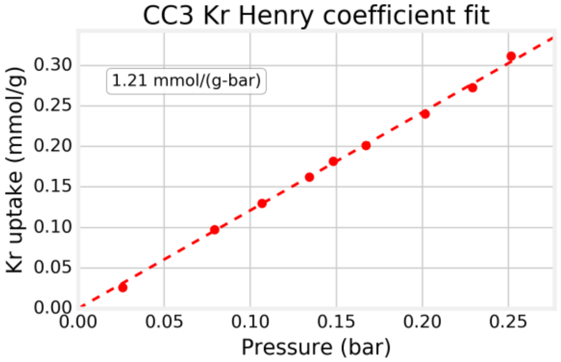

(c) **(d)**

Supplementary Figure 6. CC3. (a) Crystal structure of CC3. (b) Circles show pure component Xe (blue) and Kr (red) adsorption isotherm data at 298 K. Closed symbols are data used to identify the Henry coefficient. The dashed line shows Henry's law with the identified Henry coefficient. (c, d) same as (b) but zoomed into the Henry regime. Identified coefficient is shown in the box.

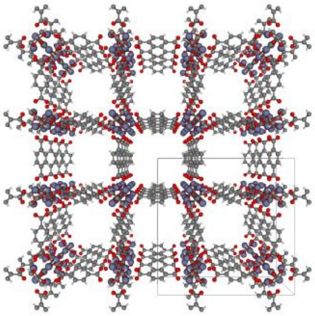

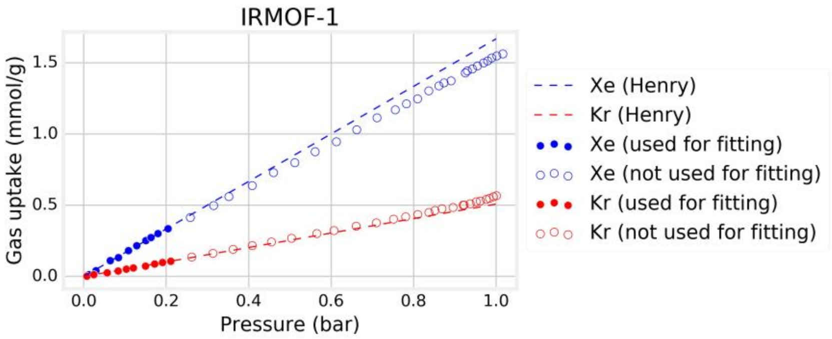

(a) **(b)**

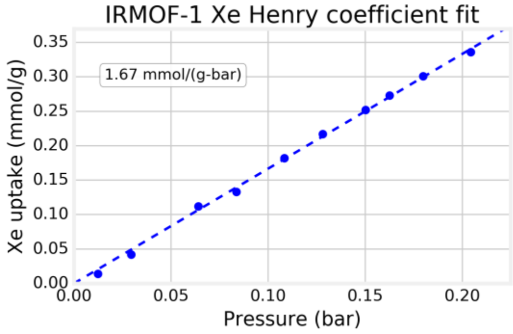

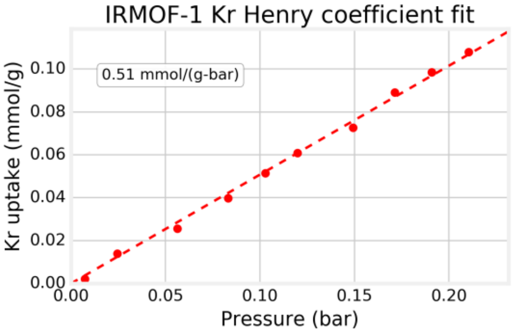

(c) **(d)**

Supplementary Figure 7. IRMOF-1. (a) Crystal structure of IRMOF-1. (b) Circles show pure component Xe (blue) and Kr (red) adsorption isotherm data at 292 K. Closed symbols are data used to identify the Henry coefficient. The dashed line shows Henry's law with the identified Henry coefficient. (c, d) same as (b) but zoomed into the Henry regime. Identified coefficient is shown in the box.

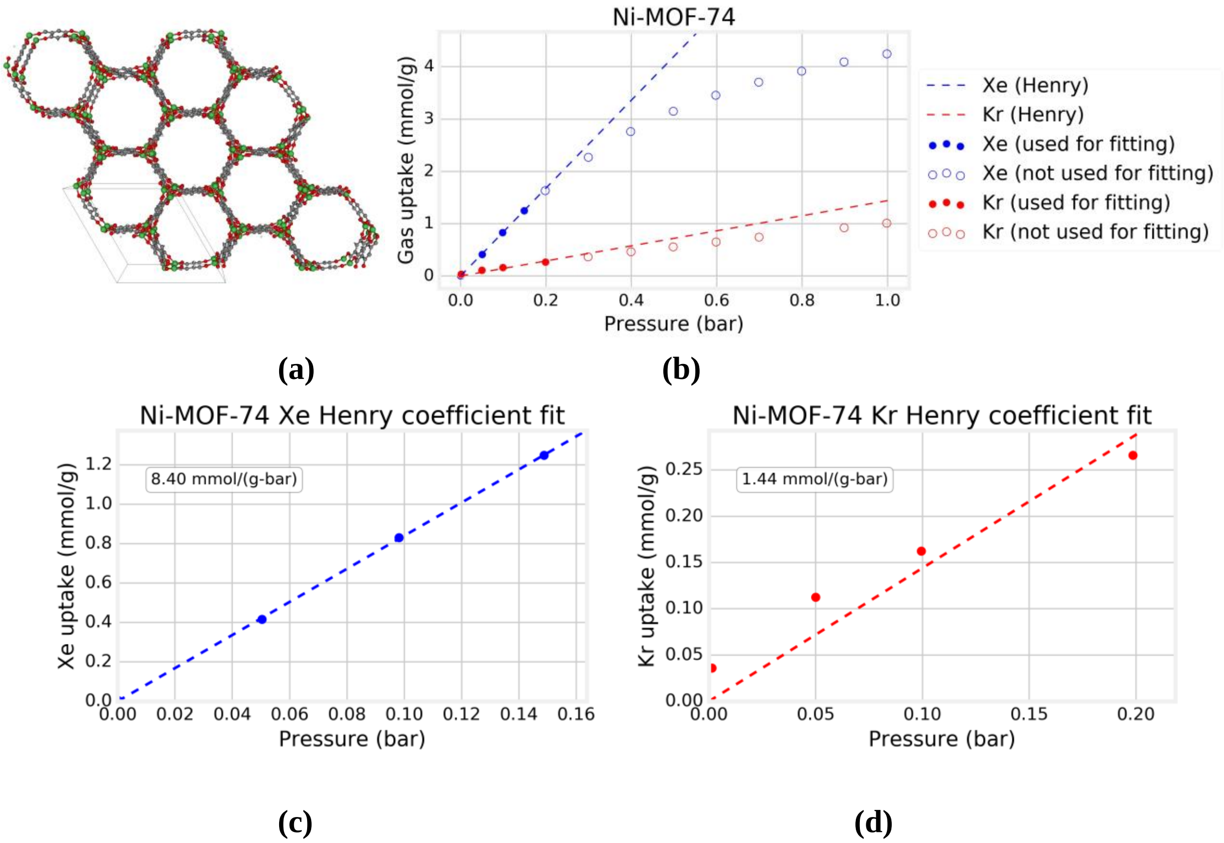

Supplementary Figure 8. NiMOF-74. (a) Crystal structure of NiMOF-74. (b) Circles show pure component Xe (blue) and Kr (red) adsorption isotherm data at 298 K. Closed symbols are data used to identify the Henry coefficient. The dashed line shows Henry's law with the identified Henry coefficient. (c, d) same as (b) but zoomed into the Henry regime. Identified coefficient is shown in the box.

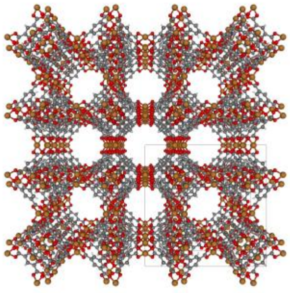

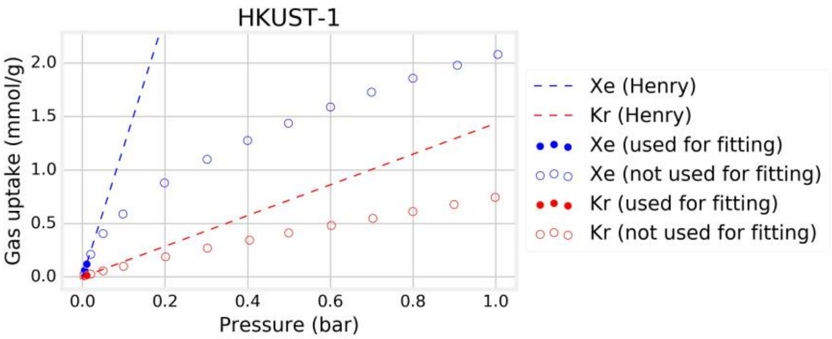

(a) (b)

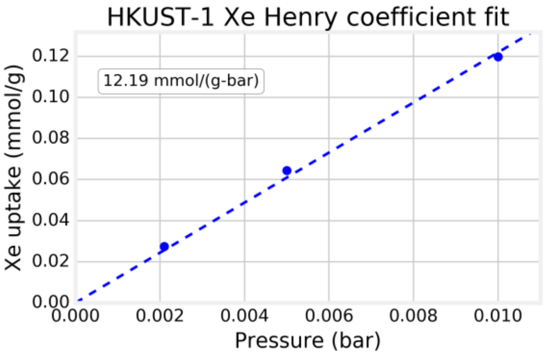

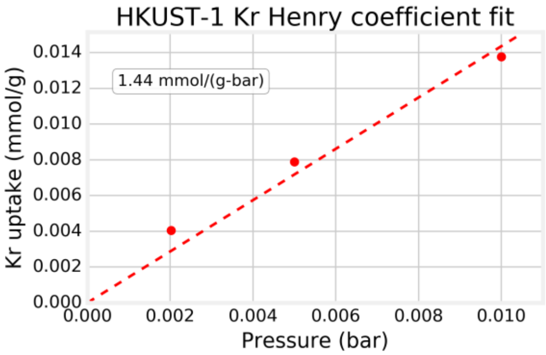

(c) **(d)**

Supplementary Figure 9. HKUST-1. (a) Crystal structure of HKUST-1. (b) Circles show pure component Xe (blue) and Kr (red) adsorption isotherm data at 298 K. Closed symbols are data used to identify the Henry coefficient. The dashed line shows Henry's law with the identified Henry coefficient. (c, d) same as (b) but zoomed into the Henry regime. Identified coefficient is shown in the box.

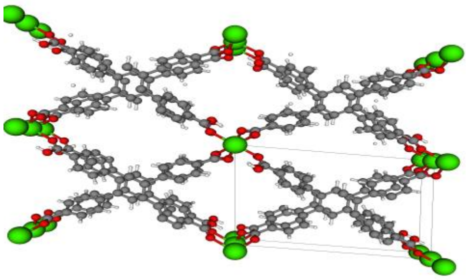

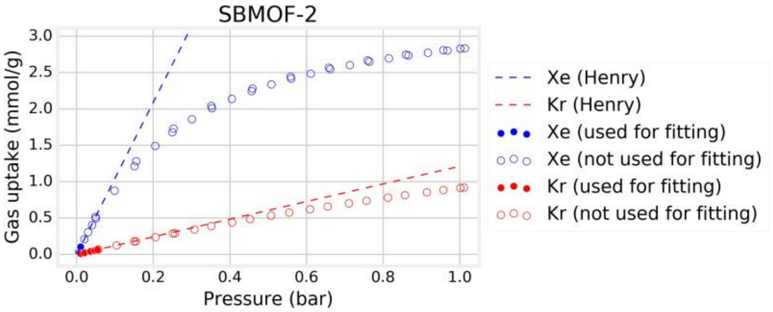

(a) **(b)**

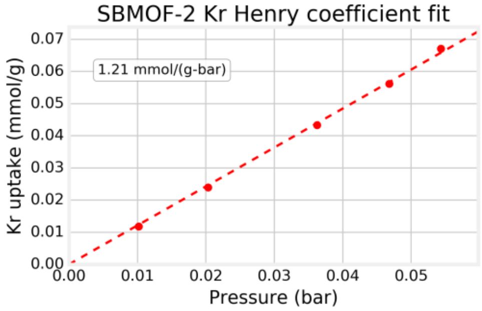

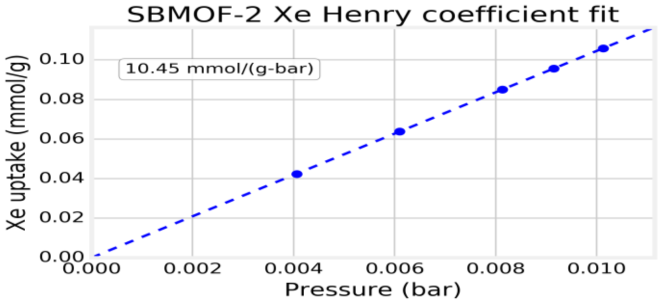

(c) **(d)**

Supplementary Figure 10. SBMOF-2. (a) Crystal structure of SBMOF-2. (b) Circles show pure component Xe (blue) and Kr (red) adsorption isotherm data at 298 K. Closed symbols are data used to identify the Henry coefficient. The dashed line shows Henry's law with the identified Henry coefficient. (c, d) same as (b) but zoomed into the Henry regime. Identified coefficient is shown in the box.

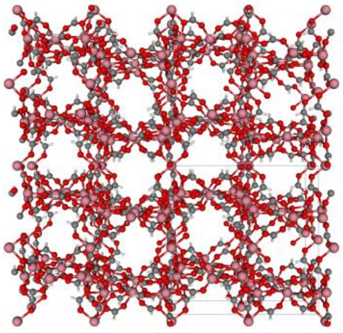

(a)

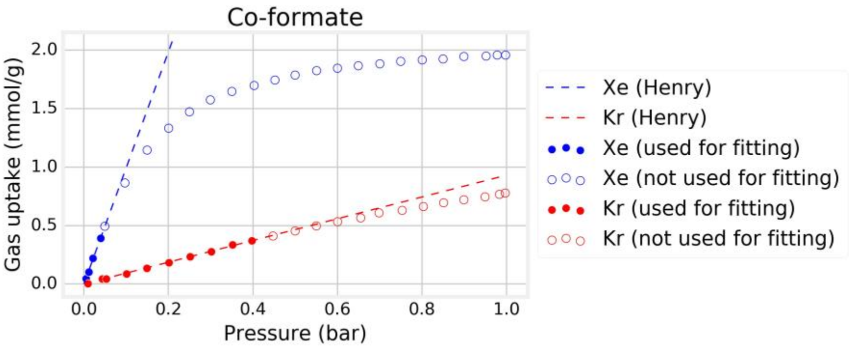

(b)

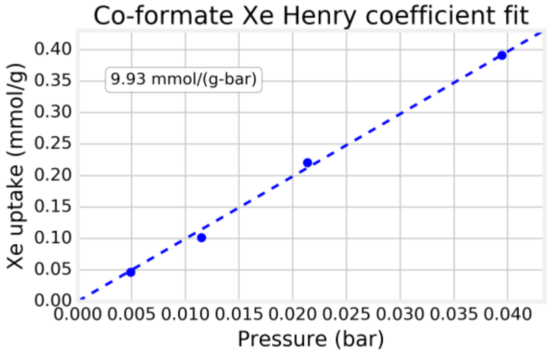

(c)

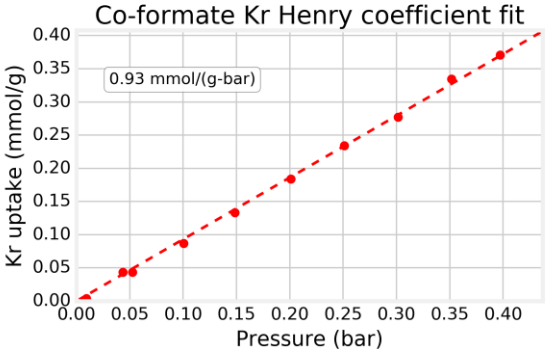

(d)

Supplementary Figure 11. Cobalt Formate. (a) Crystal structure of cobalt formate. (b) Circles show pure component Xe (blue) and Kr (red) adsorption isotherm data at 298 K. Closed symbols are data used to identify the Henry coefficient. The dashed line shows Henry's law with the identified Henry coefficient. (c, d) same as (b) but zoomed into the Henry regime. Identified coefficient is shown in the box.

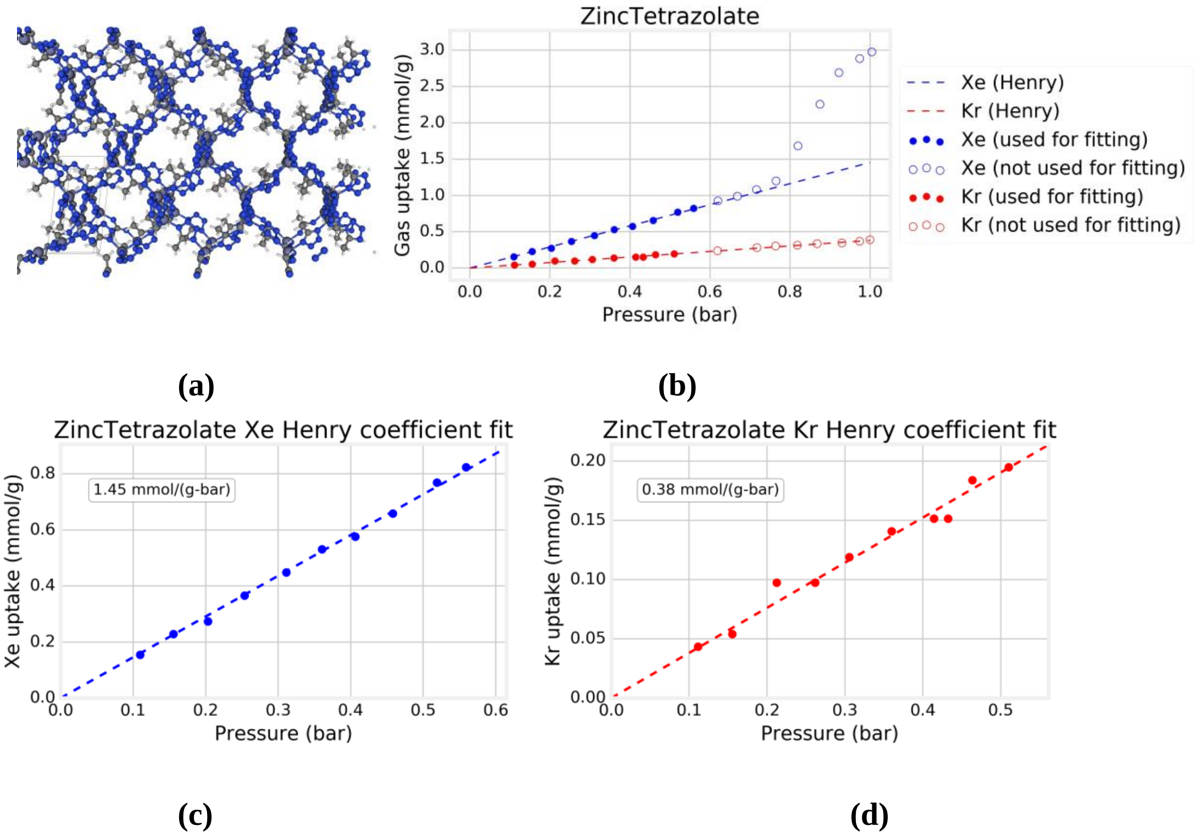

Supplementary Figure 12. Zinc Tetrazolate. (a) Crystal structure of Zinc Tetrazolate. (b) Circles show pure component Xe (blue) and Kr (red) adsorption isotherm data at 298 K. Closed symbols are data used to identify the Henry coefficient. The dashed line shows Henry's law with the identified Henry coefficient. (c, d) same as (b) but zoomed into the Henry regime. Identified coefficient is shown in the box.

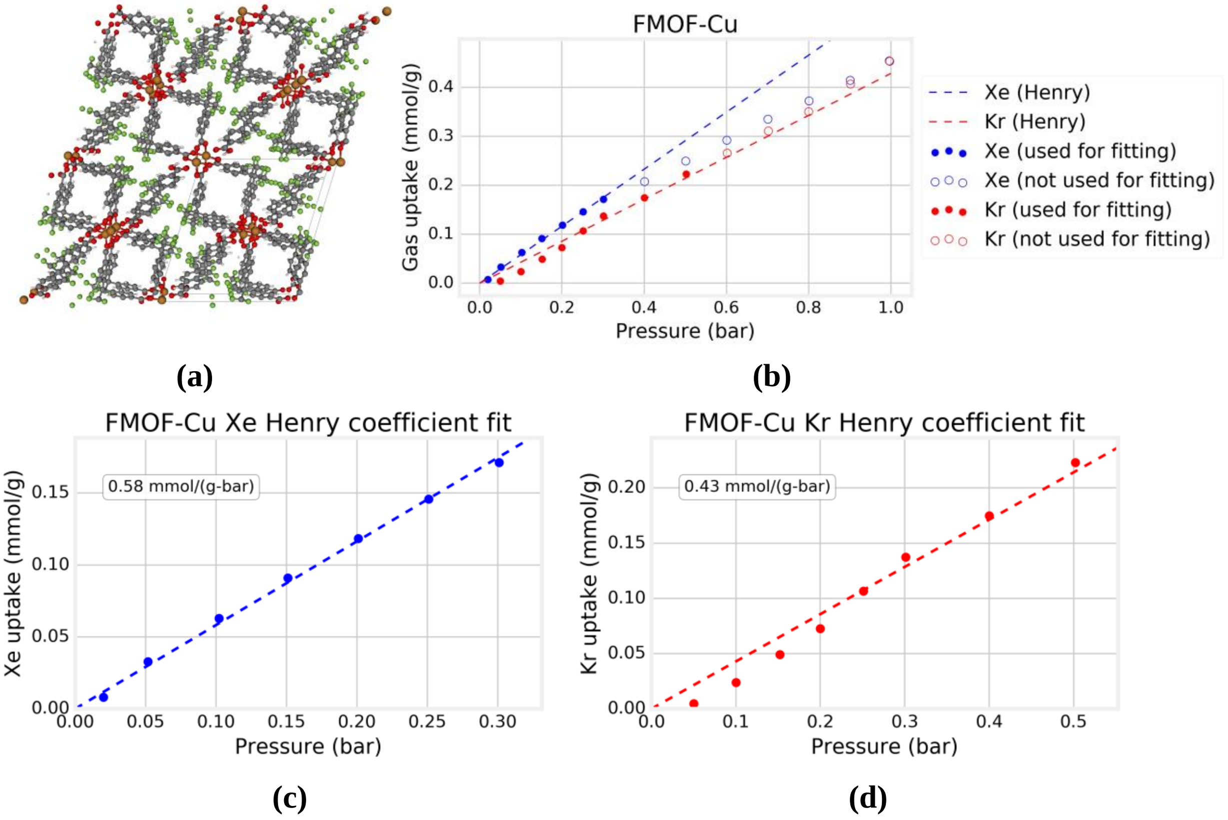

Supplementary Figure 13. FMOFCu. (a) Crystal structure of FMOFCu. (b) Circles show pure component Xe (blue) and Kr (red) adsorption isotherm data at 298 K. Closed symbols are data used to identify the Henry coefficient. The dashed line shows Henry's law with the identified Henry coefficient. (c, d) same as (b) but zoomed into the Henry regime. Identified coefficient is shown in the box.

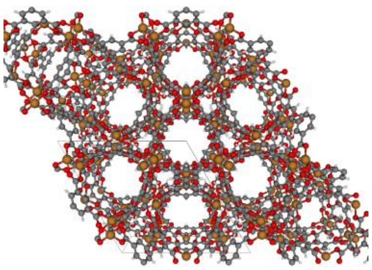

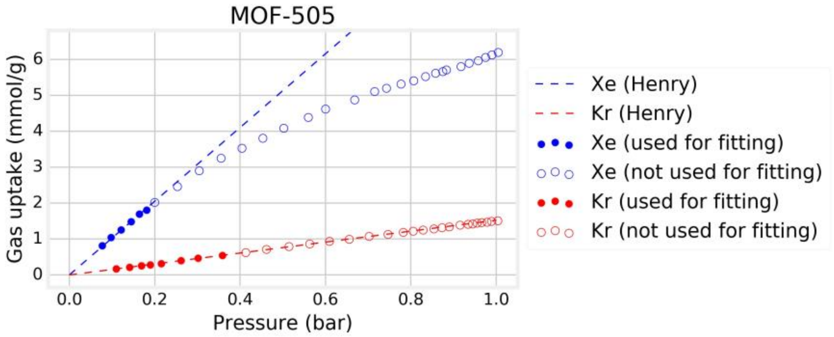

(a) **(b)**

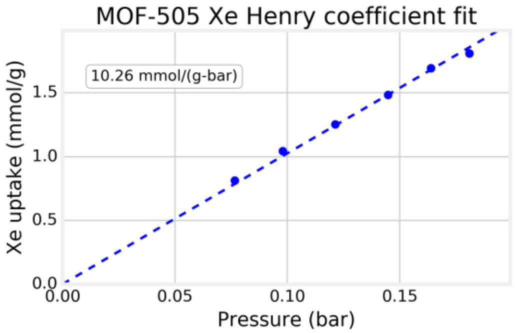

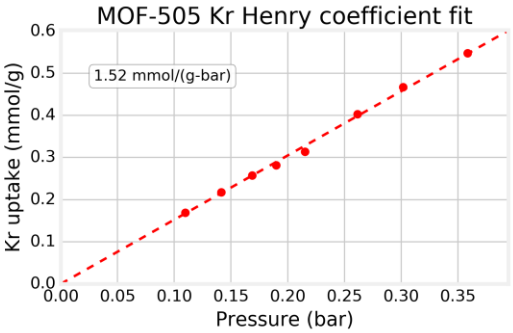

(c) **(d)**

Supplementary Figure 14. MOF-505. (a) Crystal structure of MOF-505. (b) Circles show pure component Xe (blue) and Kr (red) adsorption isotherm data at 298 K. Closed symbols are data used to identify the Henry coefficient. The dashed line shows Henry's law with the identified Henry coefficient. (c, d) same as (b) but zoomed into the Henry regime. Identified coefficient is shown in the box.

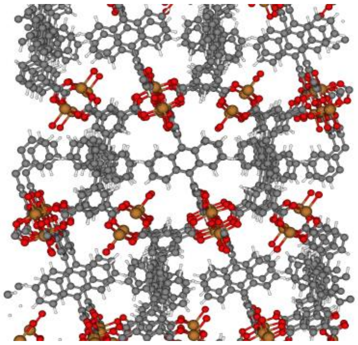

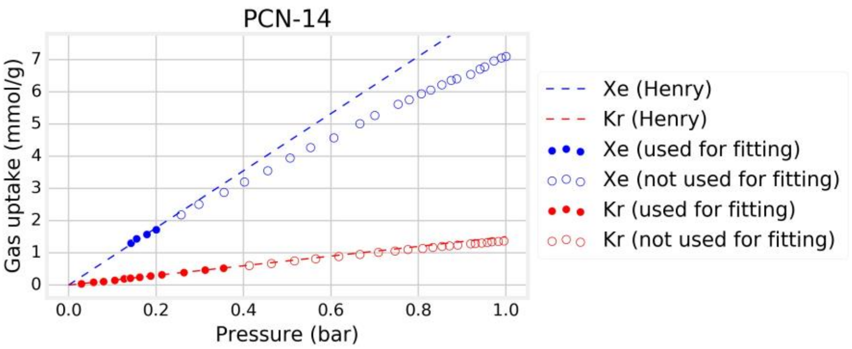

(a) **(b)**

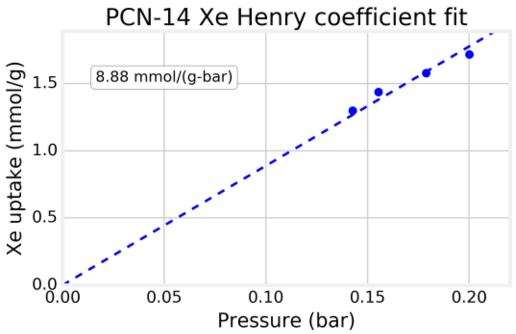

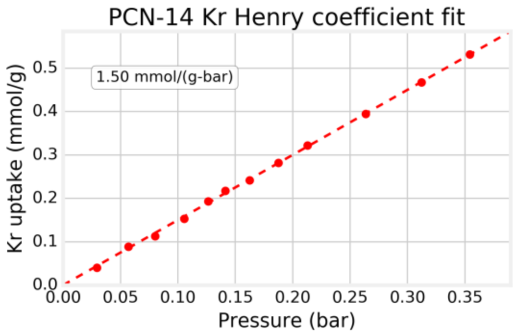

(c) **(d)**

Supplementary Figure 15. PCN-14. (a) Crystal structure of PCN-14. (b) Circles show pure component Xe (blue) and Kr (red) adsorption isotherm data at 298 K. Closed symbols are data used to identify the Henry coefficient. The dashed line shows Henry's law with the identified Henry coefficient. (c, d) same as (b) but zoomed into the Henry regime. Identified coefficient is shown in the box.

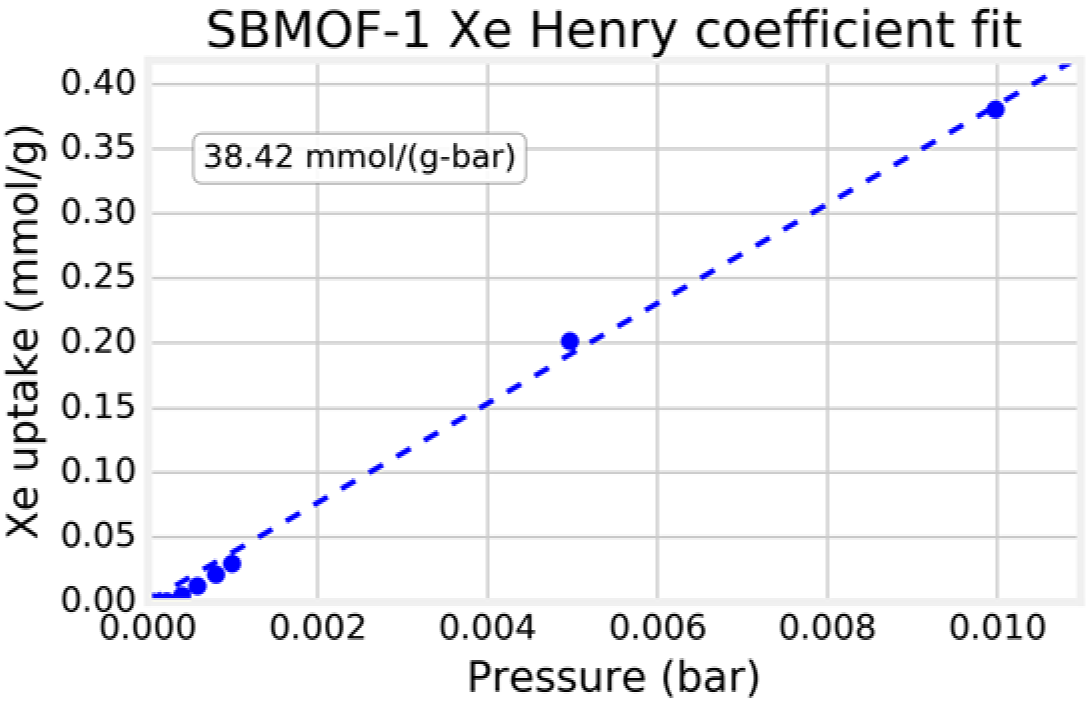

Supplementary Figure 16. The equilibrium Xe uptake capacity of SBMOF-1 at 298 K is shown as blue circles. The dashed line shows Henry's law, with the Henry coefficient identified by fitting a line of zero slope to the data shown. The resulting Henry coefficient is 38.42 mmol/(g-bar).

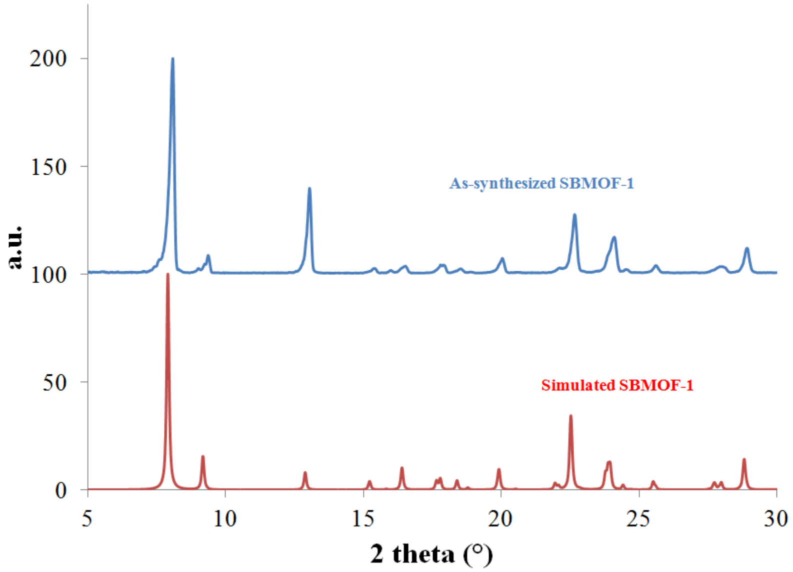

Supplementary Figure 17. The simulated (bottom) and experimental (top) powder pattern of SBMOF-1.

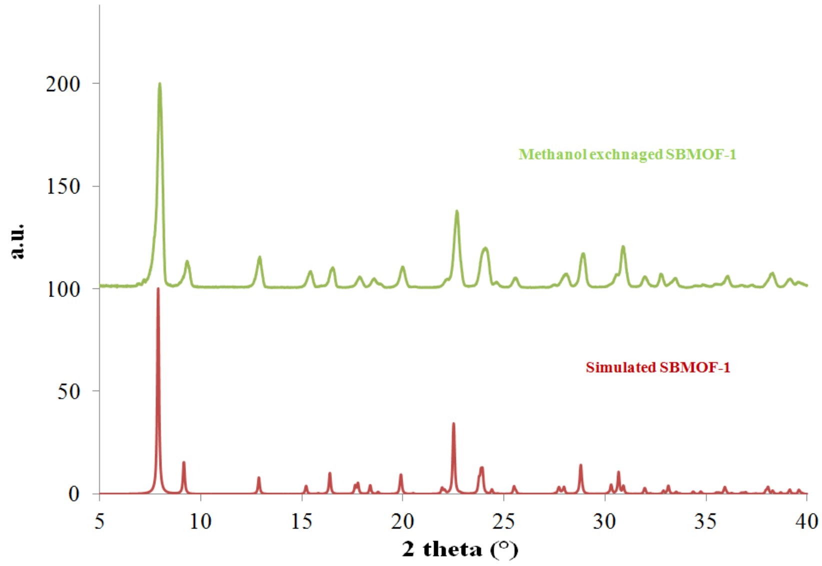

Supplementary Figure 18. The simulated (bottom) and methanol exchanged (top) powder pattern of SBMOF-1.

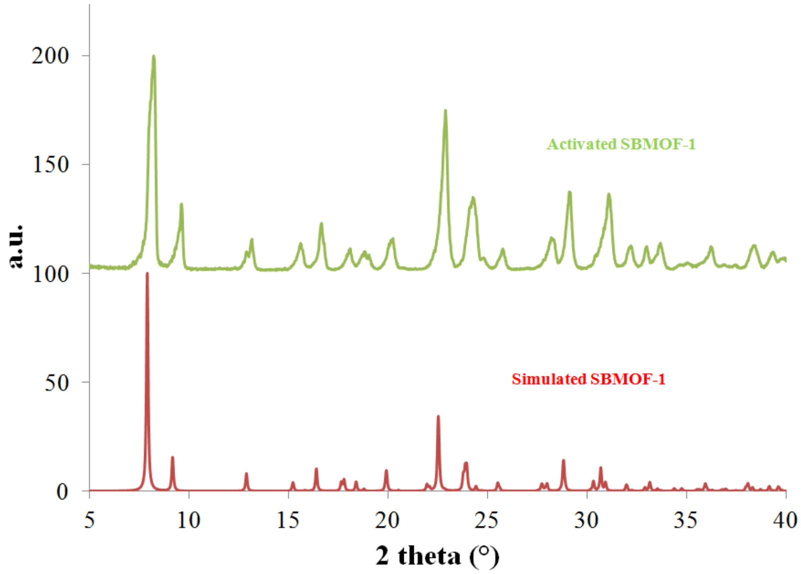

Supplementary Figure 19. The simulated (bottom) and activated (top) powder pattern of SBMOF-1

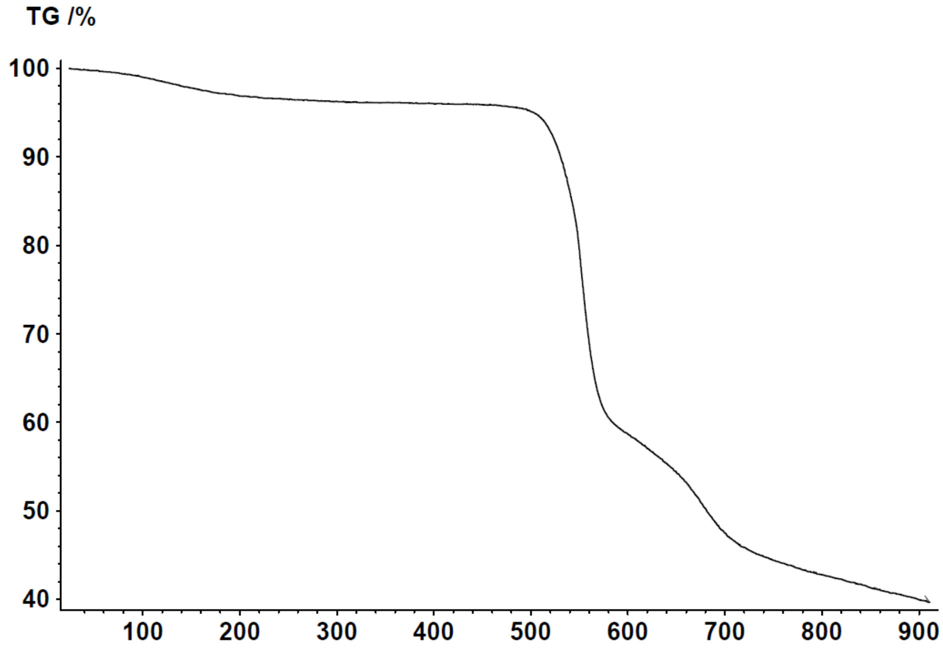

Supplementary Figure 20. Thermogravimetric pattern of as-synthesized SBMOF-1.

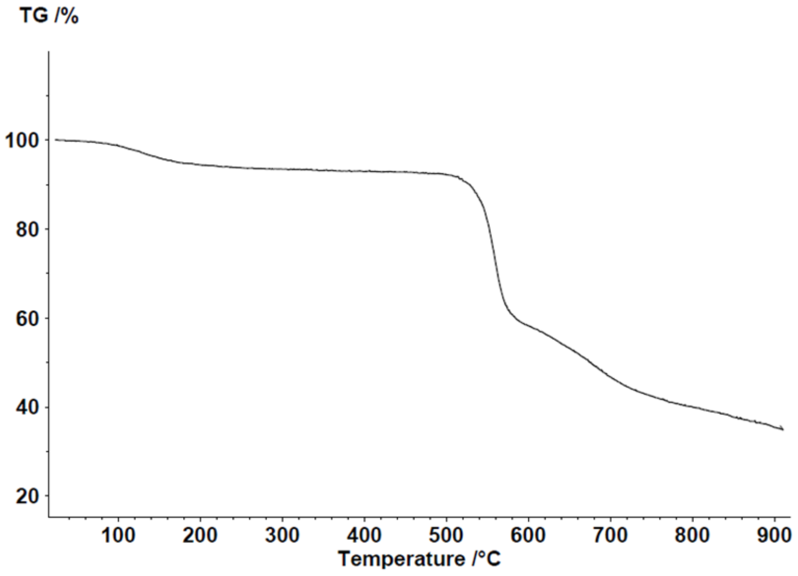

Supplementary Figure 21. Thermo-gravimetric pattern of methanol exchanged SBMOF-1

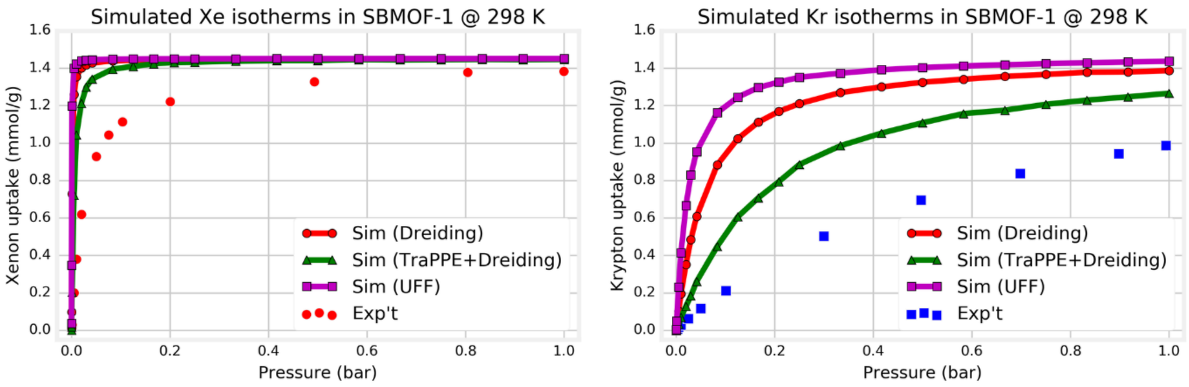

Supplementary Figure 22. Comparison between experimental and simulated pure-component Xe (left) and Kr (right) adsorption isotherms.

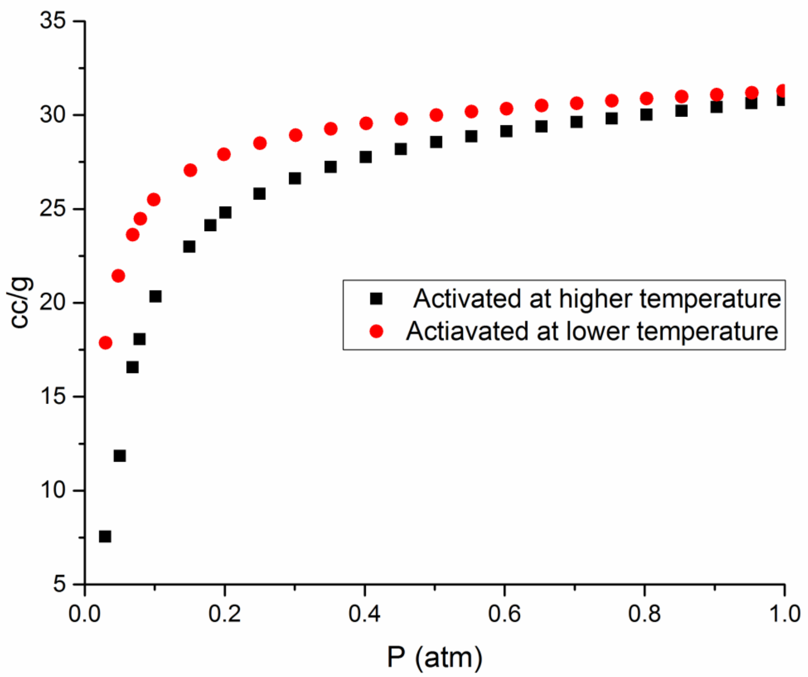

Supplementary Figure 23 The Xe adsorption isotherm of SBMOF-1, when activated at 290°C (black) and activated at 100°C (red)

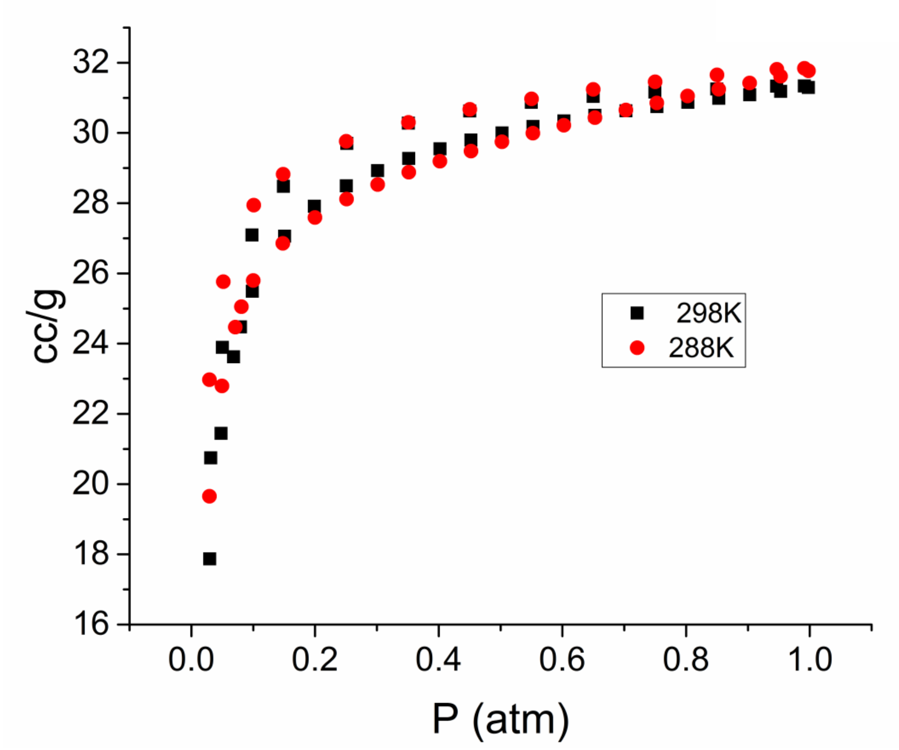

Supplementary Figure 24. The Xe adsorption isotherm (volumetric) for activated SBMOF-1 at 288K (red) and 298K (red)

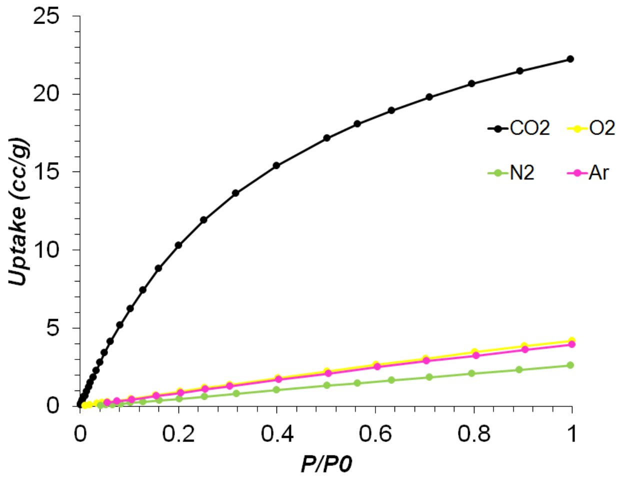

Supplementary Figure 25. Volumetric Adsorption isotherm for activated SBMOF-1 for different gases (CO_2, N_2, Ar, O_2) at 298K and 1 atm pressure.

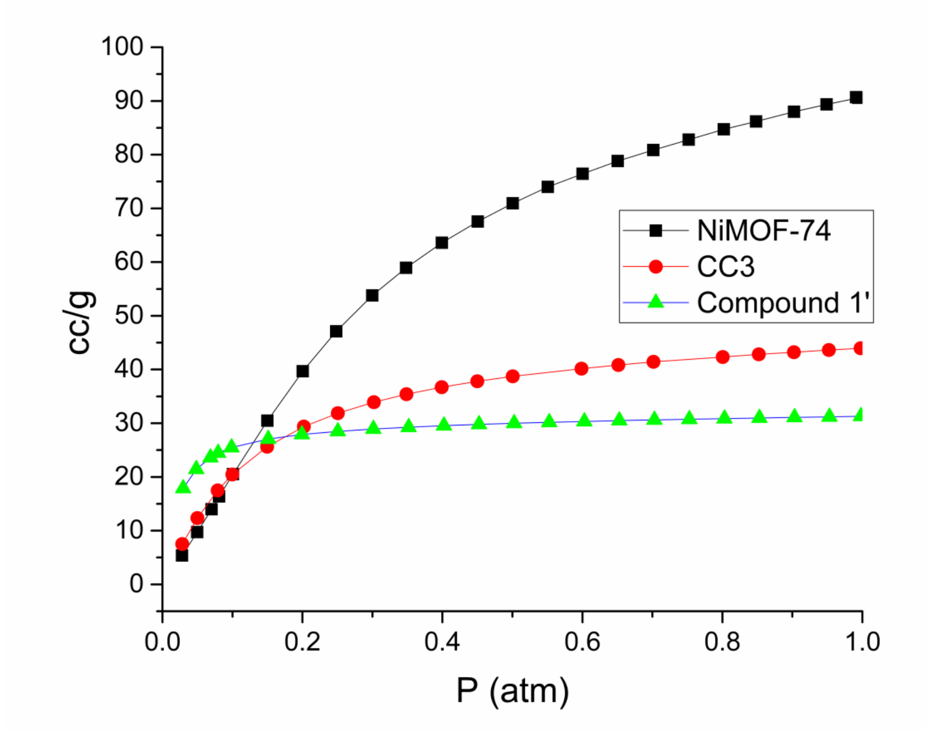

Supplementary Figure 26. Comparison of Xe adsorption for benchmark materials –NiMOF-74, CC3 and SBMOF-1. Adsorption experiments were conducted on materials synthesized at PNNL.

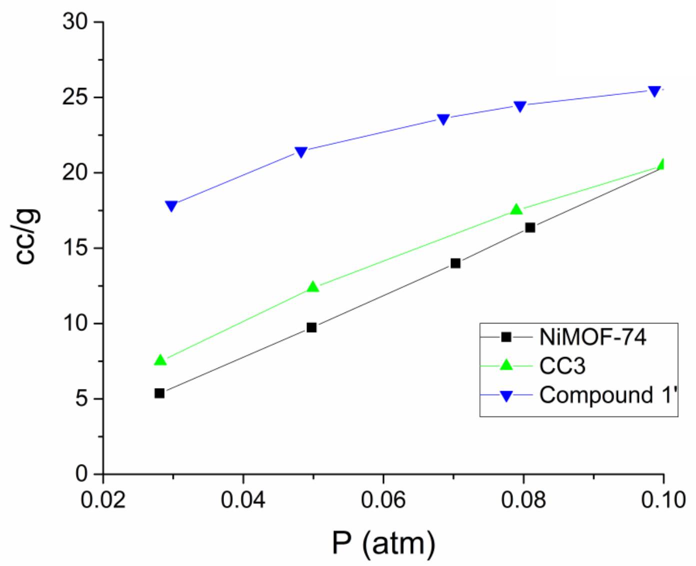

Supplementary Figure 27. Comparison of Xe adsorption for benchmark materials at 0.02-0.1 atm pressure. Adsorption experiments were conducted on materials synthesized at PNNL.

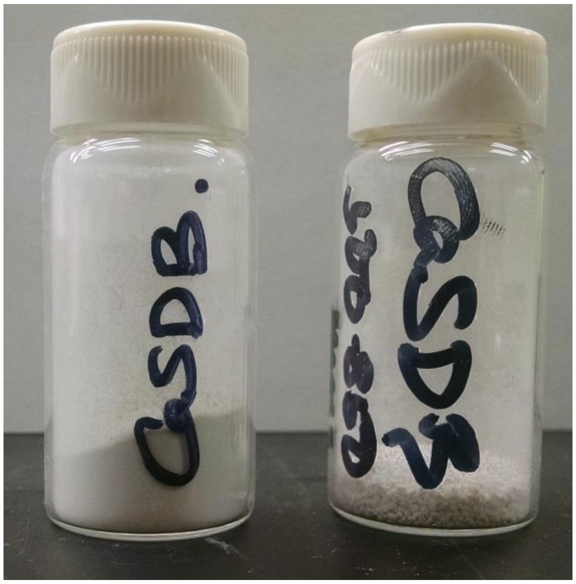

Supplementary Figure 28. Pallet (500-850 µm size) of SBMOF-1 (right), made by a mechanical sieving process (CaSDB = SBMOF-1)

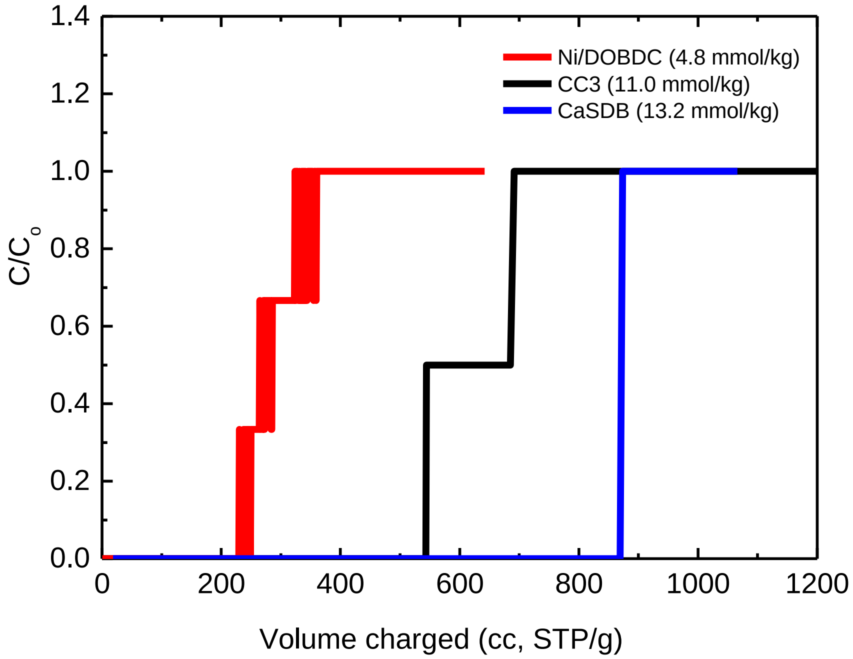

Supplementary Figure 29 Comparison of breakthrough curves for Xe (400 ppm Xe in air) adsorbed on NiMOF-74, CC3 and SBMOF-1 at 298 K. The value (C/C_0) is outlet Xe concentration divided by the inlet Xe concentration (CaSDB = SBMOF-1).

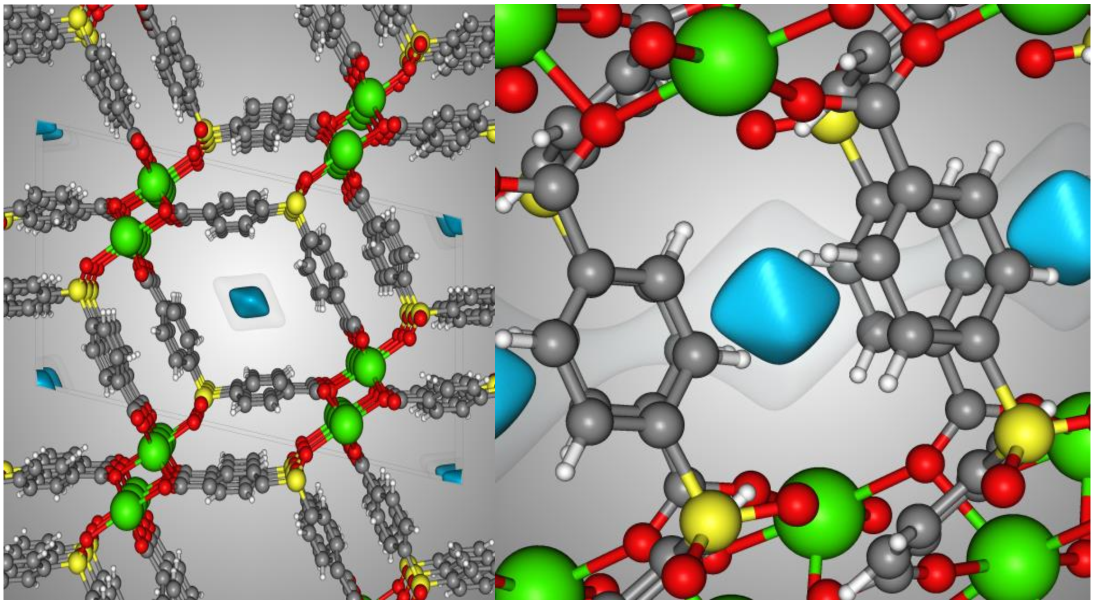

Supplementary Figure 30. Potential energy contours of a Xe atom in the SBMOF-1 structure according to the hybrid Dreiding-TraPPE-UA force field. [15 $kJmol^{-1}$: white, -32 $kJmol^{-1}$: blue]

Supplementary Table 1. Crystal data for Xe and Kr loaded SBMOF-1. The crystal data of As-synthesized SBMOF-1 is added for comparison.

	SBMOF-1	Xe@SBMOF-1	Kr@SBMOF-1
Empirical Formula	C14H8O7SCa	$C_{14}H_8O_6$SCa0.21Xe	$C_{14}H_8O_6$SCa0.15Kr
Collection Temperature (K)	298	100	100
Wavelength (λ)	0.71073	0.71073	0.71073
Space Group	$P\ 2_1/n$	$P\ 2_1/n$	$P\ 2_1/n$
a)	11.8214(3)	11.6193(5)	11.8819(3)
b (Å)	5.56730(13)	5.5667(2)	5.55550(10)
c (Å)	22.7603(6)	22.9312(10)	22.6411(5)
α (°)	90	90	90
β (°)	100.356(2)	100.897(4)	101.302(2)
γ (°)	90	90	90
Volume ($Å^3$)	1473.53(6)	1456.47	1465.55
Calculated Density(g/cm^3)	1.624	1.822	1.618

Supplementary Table 2 Lennard-Jones parameters used in the hybrid TraPPE-Dreiding model.

Atom (X)	ε (K)	σ (Å)
C-H (aromatic)	50.5	3.695
**C**(aromatic)-R	21.0	3.88
C	47.91	3.47
O	48.21	3.033
S	173.30	3.59
Ca	25.19	3.093
Xe	229.8	3.97

Supplementary Table 3. Surface density comparison of CC3 and SBMOF-1 based on geometric calculations

	Largest included sphere diameter (Å)	Crystal density (kg/m^3)	Surface area, 1.25 Å probe (m^2/cm^3)	Surface density (kg/m^2)
CC3	4.88	958	1064	9e-7
SBMOF-1	5.8	1570	766	20.5e-7

Supplementary Methods

Computational Calculation

(a) *Computational methods for screening databases of MOFs*

Here, we outline the computational methods to predict the Xe/Kr selectivity of MOFs at dilute conditions pertinent to used nuclear fuel reprocessing facility off-gas. We model the energetics of the van der Waals interactions of a Xe and Kr atom with the atoms of the MOF structures as pair-wise additive and with Lennard-Jones potentials. We take Xe-Xe and Kr-Kr Lennard-Jones parameters from Boato and Casanova. We took Lennard-Jones parameters for the atoms in the MOF structure from the Universal Force Field (UFF). Lennard-Jones parameters for the host-guest interactions then follow from Lorentz-Berthelot mixing rules. The MOF structures are held rigid throughout the simulations. We take interactions beyond a cutoff radius of 12.5 Å to be zero and implement periodic boundary conditions to mimic an infinite crystal. This molecular model is equivalent to that used in the high-throughput screening in Simon *et al*.

As Xe and Kr are present at dilute concentrations in the off-gas of used nuclear fuel reprocessing facilities, the adsorption isotherm of Xe and Kr can be modeled as:

$$N_i(p_i) = K_{H,i} p_i \qquad \mathrm{i = Xe, Kr} \quad ..(1)$$

where N_i is the amount of Xe/Kr adsorbed [moles/mass material], p_i is the partial pressure of species i [bar], and $K_{H,i}$ is the Henry coefficient of species i in the material [moles/(mass material-bar)]. The selectivity of Xe over Kr at dilute conditions is then the ratio of the Xe and Kr Henry coefficients.

The Henry coefficient is related to the spatial average of the Boltzmann factor inside the unit cell [22]

$$K_H = \langle e^{-\frac{U}{RT}} \rangle / (RT), \qquad (2)$$

where R is the universal gas constant, T is the temperature (298 K), and U is the energy of the adsorbate in the structure. We use a Monte Carlo integration algorithm (Widom particle insertions, 750 samples per $Å^3$ of material) to compute the above average in each material and

predict the Henry coefficient, using the model for the energy U described above. As each Widom particle insertion is independent of the others, this algorithm is highly amenable to parallelization; we utilized a CUDA code for GPUs to speed-up the calculation of the Henry coefficient, which enabled us to simulate Xe/Kr adsorption in large databases of materials in a reasonable time.

We computed the Henry coefficient of Xe and Kr in the following databases of MOF structures:

(i) Computation-ready, experimental (CoRE) MOFs, a database of over 4,700 computation-ready porous MOFs that have been experimentally synthesized and reported in the Cambridge Structural Database.[24]

(ii) hMOFs, a database of ~120,000 hypothetical/predicted MOFs generated computationally by snapping together molecular building blocks, much like Lego blocks or K'NEX.[25]

We visualized the distribution of selectivity among the MOFs using kernel density estimation with a Gaussian kernel (bandwidth chosen by Scott's rule) [26==] in the Stats models Python module. For the CoRE MOFs, we omitted structures that were identified to have pores that are inaccessible to Xe using a pocket-blocking algorithm. 2,867 CoRE MOFs remained.

(b) *Largest included sphere and surface area calculation*

The pores of the MOF structures were characterized using the open-source Zeo++ code, which calculates the diameter of the largest hard-sphere that can fit within a pore anywhere within a given structure and the surface area accessible to a given probe. We used the high accuracy setting and atomic radii adopted from the Cambridge Crystallographic Data Centre.

(c) *SBMOF-1 forms a denser wall of atoms than CC3*

Both SBMOF-1 and porous organic cage solid CC3 exhibit a pore diameter that is close to the optimal Xe-Xe distance in its Lennard-Jones potential, 4.5 Å (See supplementary table 1). Part of the reason that SBMOF-1 is more thermodynamically selective for Xe than CC3 is that SBMOF-1 constructs a denser wall of chemical moieties than CC3 to achieve a higher Xe binding energy, enhancing its preference for Xe. We show this here with a simple calculation. We took the ratio of the crystal density to the surface area calculated by Zeo++, which has units kg/m^2 and represents the density of atoms on the surface of the structure, *surface density*. The table below shows that SBMOF-1 has a surface density of atoms doubles of that of CC3.

(d) *Simulated Adsorption Isotherms*

We simulated the pure-component Xe and Kr adsorption isotherms in SBMOF-1 to compare with the experimental adsorption isotherms. Our molecule model is outlined in section (a). We simulated the pure-component Xe and Kr adsorption isotherms in SBMOF-1 using the Grand-canonical Monte Carlo (GCMC) algorithm. The GCMC algorithm inserts, deletes, translates, and re-grows Xe and Kr atoms inside the structure. We use 50,000 cycles to equilibrate the system and 450,000 cycles for sampling statistics, where a cycle is defined as *n* Markov chain moves, where *n* is equal to the number of adsorbates currently in the system or 20, whichever is greater. At such low pressures (<= 1 bar), we model Xe and Kr as ideal gases so that fugacity is equal to

pressure. We replicated the SBMOF-1 crystal unit cell by [3, 5, 2] in the [a, b, c] direction in order to apply the nearest image convention.

Supplementary Figure 16 shows that the Universal Force Field (UFF) and Dreiding force field overestimate the adsorption of both Xe and Kr. A similar overestimation was seen in the comparison between simulated and experimental Xe/Kr isotherms in CC3. As UFF did not accurately recapitulate the adsorption isotherm in SBMOF-1 quantitatively, this raises the question of whether it is justified to use the UFF for high-throughput screening purposes. McDaniel and coworkers compared simulated gas uptakes of CO_2 and CH_4 in 424 MOFs using *ab initio* force fields and UFF. While they observed significant quantitative differences in predicted gas uptake, they found that the UFF provided a similar *ranking* of materials as the *ab initio* force fields, justifying the use of UFF for screening purposes. The computational identification of SBMOF-1 as a highly Xe/Kr selective material and its subsequent synthesis and characterization as the highest ranked material for Xe/Kr to date is an example. A comparison between simulated and experimentally measured selectivity is found in Supplementary Figure 4; a reasonably good correlation is seen between predictions and measurements, showing that simulations using UFF can reasonably rank materials on the basis of their Xe/Kr selectivity. To more accurately describe the interactions in the SBMOF-1 structure, we modeled the aromatic ring in SBMOF-1 using the TraPPE-UA parameters for toluene and took the remaining parameters from Dreiding, creating a hybrid Dreiding- TraPPE-UA force field. See supplementary table 2 above. We utilized this hybrid Dreiding-Trappe-UA force field to visualize the potential energy contours in SBMOF-1 since the simulated isotherm using this force field is closer to the experiment, as supplementary figure 16 shows.

(e) *Snapshots of Xe and Kr during the Grand-Canonical Monte Carlo Simulation*

During the Grand-canonical Monte Carlo simulation of Xe and Kr adsorption with the hybrid Dreiding-TraPPE-UA force field, we recorded 10,000 snapshots of the system (positions of Xe, Kr inside the pores of SBMOF-1) at 1 bar pure component and 298 K. Fig. 5 in the main text visualizes the spatial probability density of Xe and Kr adsorbates inside the structure, which agrees with the Xe adsorption site determined by the X-ray diffraction experiments.

(f) *Potential energy contour visualization*

To visualize the potential energy of a Xe atom inside the SBMOF-1 structure, we used the hybrid Dreiding-TraPPE-UA force field to compute the potential energy on a three-dimensional grid of points superimposed on the unit cell of the crystal. Then, we visualized the potential energy contours using the VisIt visualization tool.

(g) *Consistency between breakthrough capacity and adsorption isotherm*

In the breakthrough experiments, at the point where Xe breaks through the SBMOF-1 adsorbent bed, we observed that the bed captured ~13.2 mmol Xe/kg. Here, we compare this to the equilibrium uptake of Xe in SBMOF-1 using Henry's law. We fit a line to the Xe adsorption

isotherm in SBMOF-1 to estimate the Henry coefficient and predicted that, in a gas mixture with 400 ppm Xe, the equilibrium uptake of Xe is 15.37 mmol/kg, close to the Xe capacity in the breakthrough measurements. We expect capacity to be lower than the equilibrium uptake because of diffusion limitations; this deviation by ~15% suggests that the diffusion limitations within SBMOF-1 are not severe.

(h) *Literature review of high-throughput screenings for Xe/Kr separations*

A few studies in the literature used molecular simulations to screen MOFs for Xe/Kr separations. The context considered for these screening studies was a 20/80 mol% Xe/Kr mixture at 1 bar and 298 K. This mixture is pertinent to the conventional method to obtain pure Xe and pure Kr as a byproduct of the cryogenic distillation of air. Ryan and coworkers first screened a set of eight MOFs reported in the literature. Later, Sikora and coworkers screened a database of 127,000 hypothetical MOF structures. Van Heest and coworkers screened a larger database of experimentally reported MOFs. SBMOF-1 was not reported in this study as a top performer likely because the simulation-ready database of experimentally reported MOFs was incomplete and SBMOF-1 was only recently reported. Finally, Simon and coworkers used a combination of machine learning and molecular simulations to screen databases of nanoporous materials amounting to over half a million. SBMOF-1 was found to be the most Xe-selective material in this study. However, we found that the best materials for separating a 20/80 mol% Xe/Kr mixture at 1 bar and 298 K are generally different than those for capturing Xe present in air at dilute conditions. Thus, in this work, we performed a high-throughput screening of MOFs at dilute conditions that pertain to UNF reprocessing. Remarkably, SBMOF-1 still turned out to be the highest ranked material among the experimental MOFs.

Synthesis

SBMOF-1 was originally synthesized using a previously published literature procedure by Banerjee and coworkers. Starting materials include calcium chloride ($CaCl_2$, 96%, Acros-Organics), 4,4'-sulfonyldibenzoic acid (4,4'-SDB, 98%,Sigma-Aldrich) and ethanol (95%, Fisher-Scientific) and were used without purification. In a typical synthesis, a mixture of 0.6 mmol of $CaCl_2$ (0.074 g) and 0.6 mmol of 4,4'-SDB (0.198 gram) were added in 10 ml of ethanol and stirred for ~2 hours to achieve homogeneity [molar ratio of metal chloride : ligand: solvent = 1:1:380]. The resultant solution was heated at 180°C for 3 days. Colorless, needle shaped crystals were recovered as product and washed with ethanol (yield: 45% based on $CaCl_2$, 0.1 gram). In several cases, recrystallized organic linker was found to be a side product, in those cases, the product was washed with DMF to ensure complete removal of the linker. For scale up, 1.44 gram of $CaCl_2$ (13 mmol) and to 3.98 gram of 4,4'-SDB (13 mmol) were added to 120 ml of ethanol and stirred for ~2 hours to achieve homogeneity [molar ratio: 1:1:156]. The well-mixed solution was then transferred to three 100 ml Teflon lined stainless steel Parr autoclaves and heated for 2 days at 180°C. The product was obtained as white powder and was washed by ethanol (3x times, 50 ml), followed by drying under vacuum (yield: 2.2 gram, 50% based on

$CaCl_2$). The as-synthesized material was then exchanged with methanol (3x times, 50 ml) for a total period of 3 days. After that, the material was activated at 100°C under vacuum for 12 hours.

Effect of Activation Temperature

The original synthesis paper by us reported an activation temperature of 290°C for 12 hours. At this temperature, the activated material has a different denser phase. Both the as-synthesized and the activated phase at 290°C (Act_{290}) have the same space group ($P2_1/n$), but different packing of the organic linker. In as-synthesized phase, the linkers of the same channel are packed in a parallel manner, but after activation they are turned by 45° to each other. As a result, the volume of the channel is smaller, changing the overall porosity from 18% to 16%. On the contrary, the activated phase at 100°C (Act_{100}) does not show such ring rotation. We postulate that this is because there is not enough supply of activation energy in the 2nd case for rings to rotate. Although there is not much difference in total capacity for two differently activated materials (i.e. at 100°C- Act_{100} and 290°C- Act_{290}), the difference at lower pressure (~30 mbar) is dramatic, with Act_{100} having 2.5 times more Xe uptake capacity than Act_{290}. Due to the ring rotation, the pore size of Act_{290} is less than optimal for Xe atom to fit in and thus a ring rotation back to Act_{100} (or as-synthesized) is necessary. This is also supported by the fact that when we performed a single crystal XRD experiment on Xe loaded Act_{290}, the rings were found to be back to their original position. Interestingly, such behavior was not observed for Kr loaded structures, confirming our hypothesis.

Breakthrough measurement

Low concentration Xe (400 ppm) and Kr (40 ppm) mixture in air breakthrough experiments were carried out using a dynamic sorption analyzer (ARBC, Hiden Analytical Ltd., Warrington, U.K.). To minimize pressure drop and to prevent potential contamination of the main gas pipelines, a pellet sample of SBMOF-1 was prepared following a two-step procedure. First, a powder sample was pressed into a disk under 75 MPa for 3 min. The disk was then carefully broken up using a pestle and mortar and the fragments were sieved for 20–35 mesh (500-850 μm) pellets (supplementary figure 30). The two-step procedure was repeated to make more pellets where necessary. The cage pellets were packed into an adsorption bed for the breakthrough experiment. With reference to the ARBC system illustrated in a previous paper, the gases were introduced through the bottom inlet of the adsorption bed. The adsorption bed was held between two layers of quartz wool and two sample holders, with frit gaskets installed at both the top and bottom ends of the adsorption bed to further prevent any potential powder contamination of the pipelines. For the separation of Xe (400 ppm in air) and Kr (40 ppm in air) at 298 K, a total flow rate of 20 cm^3 STP min^{-1} and a total pressure of 1 bar were used. Prior to the breakthrough experiment, the pellet sample was degassed by heating at 373K *in situ* under a helium purge for 12 h. The activated sample weight (1.48 gram) was determined immediately after unloading the activated sample and the ideal gas law was used to calculate the moles of gas adsorbed by activated SBMOF-1. The breakthrough experiments on NiMOF-74 and CC3 were conducted using similar setup.

Based on the mass balance, the gas adsorption properties can be determined as follows:

$$q = \frac{C_0 V t_s}{22.4\ W} \ldots (3)$$

$$t_s = 1 - \frac{F}{F_0} t \ldots (4)$$

where t_s is the stoichiometric time (min), C_0 is the feed gas concentration, F_0 and F are the inlet and outlet gas molar flow rates respectively, q is the equilibrium adsorption capacity of gas (mmol g^{-1}), t is the adsorption time (min) which is from time zero to time when equilibrium is reached, V is the volumetric feed flow rate ($cm^3\ min^{-1}$) at standard temperature and pressure (1 atm and 273K) and W is the weight of the activated adsorbent (g).

The selectivity was then calculated according to the equation:

$$S_{Xe/Kr} = \frac{x_{Xe}}{x_{Kr}} \Big/ \frac{y_{Xe}}{y_{Kr}} \ldots (5)$$

where *Xe* and *Kr* are the mole fractions of gases Xe and Kr in the adsorbed phase and *Xe* and *Kr* are the mole fractions of gases Xe and Kr in the bulk gas phase.

Single crystal X-ray diffraction

The activated crystals of SBMOF-1 were placed in a three-neck conical flask. The crystals were exposed to Xe and Kr respectively for approximately ~10 minutes. After that, the crystals were coated with Paratone® oil, while maintaining the flow of the respective gases. The crystals were analyzed under an optical microscope, fitted with polarizing optics, and high-quality single-crystals were chosen for structural analysis. Reflection of Xe and Kr loaded SBMOF-1 [Xe@SBMOF-1 and Kr@SBMOF-1 respectively] were collected using a four circle kappa Oxford Gemini diffractometer equipped with an Atlas detector (λ = 0.71073) at 100K. The raw intensity data were collected, integrated and corrected for absorption effects using CrysAlis PRO software. Data sets were corrected for absorption using a multi-scan method, and structures were solved by direct methods using SHELXS-97 and refined by full-matrix least squares on F^2 with SHELXL-97. Atoms belonging to the framework were located first, and refined anisotropically. Hydrogen atoms were added to the structure model using geometrical constraints (HFIX command). After obtaining satisfactory model of the framework, gas atoms (Xe, Kr) were located from Fourier difference map and refined anisotropically with the occupancy refined.
